# The Fight against Cancer by Microgravity: The Multicellular Spheroid as a Metastasis Model

**DOI:** 10.3390/ijms23063073

**Published:** 2022-03-12

**Authors:** Daniela Grimm, Herbert Schulz, Marcus Krüger, José Luis Cortés-Sánchez, Marcel Egli, Armin Kraus, Jayashree Sahana, Thomas J. Corydon, Ruth Hemmersbach, Petra M. Wise, Manfred Infanger, Markus Wehland

**Affiliations:** 1 Department of Microgravity and Translational Regenerative Medicine, Medical Faculty, University Hospital Magdeburg, Otto von Guericke University, Universitätsplatz 2, 39106 Magdeburg, Germany; herbert.schulz@med.ovgu.de (H.S.); marcus.krueger@med.ovgu.de (M.K.); jose.cortes@ovgu.de (J.L.C.-S.); armin.kraus@med.ovgu.de (A.K.); manfred.infanger@med.ovgu.de (M.I.); markus.wehland@med.ovgu.de (M.W.); 2 Clinic for Plastic, Aesthetic and Hand Surgery, Medical Faculty, University Hospital Magdeburg, Otto von Guericke University, Leipziger Straße 44, 39120 Magdeburg, Germany; 3 Research Group ‘Magdeburger Arbeitsgemeinschaft für Forschung unter Raumfahrt- und Schwerelosigkeitsbedingungen’ (MARS), Otto von Guericke University, Universitätsplatz 2, 39106 Magdeburg, Germany; 4 Department of Biomedicine, The Faculty of Health, Aarhus University, Ole Worms Allé 4, 8000 Aarhus, Denmark; jaysaha@biomed.au.dk (J.S.); corydon@biomed.au.dk (T.J.C.); 5 Space Biology Group, Institute of Medical Engineering, Lucerne University of Applied Sciences and Arts, 6002 Hergiswil, Switzerland; marcel.egli@hslu.ch; 6 Department of Ophthalmology, Aarhus University Hospital, Palle Juul-Jensens Blvd. 99, 8200 Aarhus, Denmark; 7 Gravitational Biology, Institute of Aerospace Medicine, German Aerospace Center, Linder Höhe, 51147 Cologne, Germany; ruth.hemmersbach@dlr.de; 8 The Saban Research Institute, Children’s Hospital Los Angeles, University of Southern California, 4650 Sunset Blvd., Los Angeles, CA 90027, USA; wisepetra@gmail.com

**Keywords:** cancer stem cells, thyroid cancer, breast cancer, prostate cancer, liver cancer, colorectal cancer, microgravity, multicellular spheroids, organoids, clinostat, random positioning machine, rotating wall vessel, spaceflight, omics studies

## Abstract

Cancer is a disease exhibiting uncontrollable cell growth and spreading to other parts of the organism. It is a heavy, worldwide burden for mankind with high morbidity and mortality. Therefore, groundbreaking research and innovations are necessary. Research in space under microgravity (µ*g*) conditions is a novel approach with the potential to fight cancer and develop future cancer therapies. Space travel is accompanied by adverse effects on our health, and there is a need to counteract these health problems. On the cellular level, studies have shown that real (r-) and simulated (s-) µ*g* impact survival, apoptosis, proliferation, migration, and adhesion as well as the cytoskeleton, the extracellular matrix, focal adhesion, and growth factors in cancer cells. Moreover, the µ*g*-environment induces in vitro 3D tumor models (multicellular spheroids and organoids) with a high potential for preclinical drug targeting, cancer drug development, and studying the processes of cancer progression and metastasis on a molecular level. This review focuses on the effects of r- and s-µ*g* on different types of cells deriving from thyroid, breast, lung, skin, and prostate cancer, as well as tumors of the gastrointestinal tract. In addition, we summarize the current knowledge of the impact of µ*g* on cancerous stem cells. The information demonstrates that µ*g* has become an important new technology for increasing current knowledge of cancer biology.

## 1. Introduction

Since the beginning of the age of space travel in the 1960s, the question of if and how microgravity (µ*g*) influences whole organisms and cells has been one major focus in the field of space medicine and related disciplines. Over the years, it has become clear that µ*g* can induce a multitude of health risks in astronauts, including bone loss, muscle atrophy, cardiac atrophy, visual impairment, increased intracranial pressure, cardiovascular complications, skin problems, increased infection risk, impaired immune system, motion sickness, and orientation problems (besides the radiation-induced effects during space travel, such as an increased risk for cancer or cataracts) [1,2,3,4,5,6,7,8].

Furthermore, it became apparent that humans, microorganisms, and cultured mammal cells go through µ*g*-induced changes. For example, the virulences of *Listeria monocytogenes*, methicillin-resistant *Staphylococcus aureus*, *Enterococcus faecalis*, and *Candida albicans* were shown to decrease in microgravity [9]. In mammalian cells, µ*g* is able to disrupt the cytoskeleton [10], which is thought to be a major factor for cellular graviperception [11]. It was also found that cell growth under µ*g* could induce a spontaneous assembly of three-dimensional (3D)-tissue constructs from cell monolayers, ranging from more amorphous multicellular spheroids (MCS) to more complex vascular intima-like structures [12,13,14,15,16,17,18].

This comprehensive review will focus on the impact of µ*g* on cancer cells, which are of particular interest due to possible implications and discoveries, which might help to develop new anti-cancer drugs in the future.

## 2. Platforms for Microgravity-Based Research

### 2.1. Ground-Based Facilities: Simulators of Weightlessness on Earth

Earth-bound µ*g*-simulators, so-called ground-based facilities, can hardly replace experimentation under r-µ*g* conditions. However, they can provide valuable insight into the effects of gravitational conditions, which are different from Earth’s gravitational field (1*g*). Weightlessness conditions can be simulated to some extent on the ground. In the case of humans, bedrest with 6° head-down tilt has become a suitable model to study muscle and bone loss, fluid shift, and consequences of increased intracranial pressure, parameters all associated with health-related problems which can be observed during long-term spaceflight [19]. Understanding the underlying mechanisms aims to develop countermeasures with respect to human health on Earth. Hindlimb suspension and mechanical unloading are approaches in animal physiology to study the potential influence of weightlessness on specific organ and system functions [20].

This review focuses on cellular and tissue studies under altered gravity conditions. For this kind of studies, various platforms have been designed to mimic weightlessness conditions, i.e., creating an environment for cells that prevents sedimentation and the registration of the unidirectional gravitational stimulus comparable to the situation in real microgravity. Perception of a stimulus needs specific sensors and is characterized by distinct sensitivities and the ability to register the specific strength of a stimulus and thus the existence of thresholds [21]. Understanding potential side effects and the physics behind the simulation approach are challenges and prerequisites to reduce the risk of misinterpreting experimental results.

In this context, magnetic levitation as a simulation approach for µ*g* studies of living systems on Earth is excluded from this review. Experiments in high magnetic fields have proven to be inaccurate simulations of µ*g*. Objects can be levitated, but the influence of the strong magnetic fields of up to 30 Tesla is immense, and itself triggers responses that mask the effects known from experiments in r-µ*g* [22].

Rotation-based devices are described in the following. This approach can effectively mimic free-fall/µ*g* for small biological objects, thereby providing insights and predictions about how cells, tissues, and organisms react to altered gravity conditions. To what degree an object really experiences “weightlessness” and is therefore exposed to a high-quality simulation vastly depends on the sensitivity of its gravity perception mechanism.

Clinostats, Random Positioning Machines (RPMs), and Rotating Wall Vessels (RWVs) are frequently used as standard devices for gravitational biology research delivering valuable reference data for experiments on r-µ*g* platforms, including the International Space Station (ISS). Due to a number of side effects and the individual responses of microorganisms, cells, organs, and small organisms to the various simulation methods, conclusions should not be drawn without subsequent verification through results from experiments under r-µ*g* conditions. Additionally, comparative approaches using different devices are useful to increase our understanding and validate the simulation efforts.

**Figure 1:**
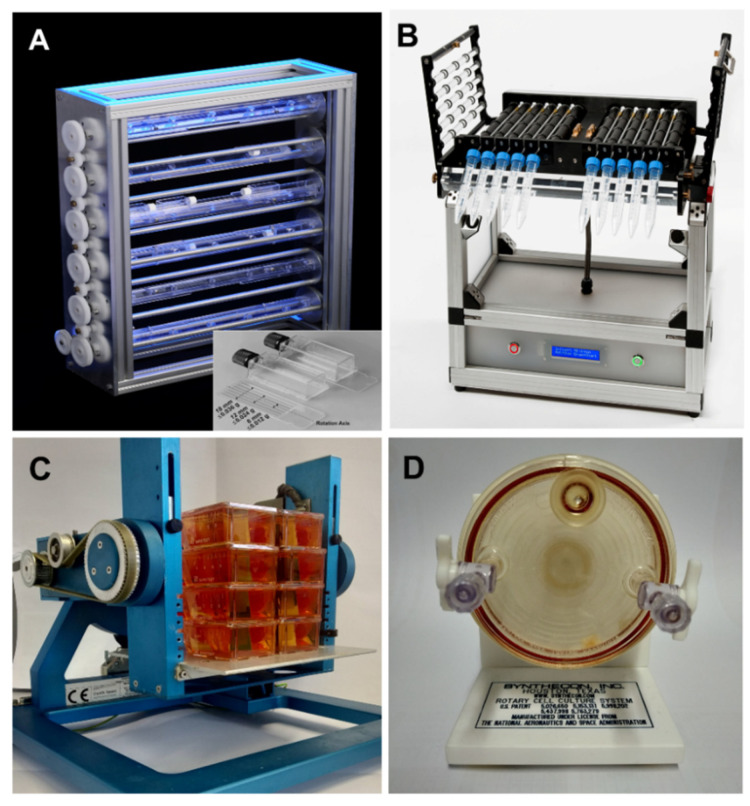
Ground-based facilities: (**A**) 2D clinostat for adherent cells in slide flaks (Insert in A demonstrates residual accelerations depending on speed of rotation (here 60 rpm) and diameter) or (**B**) for suspended cells in pipettes. (**C**) Random Positioning Machines equipped with slide flasks (mL) for thyroid cancer cells. (**D**) NASA-developed Rotating Wall Vessel.

Clinostats are tools that mimic the effects of µ*g* to varying degrees by rotating samples around one or two axes of rotation (for recent overviews, see [23,24,25]). A 2D clinostat is characterized by just one rotation axis. It rotates continuously with constant speed and direction and thereby rotates exposed systems perpendicular to the direction of the Earth’s gravity vector. Small-sized samples are located as close as feasible to the axis of rotation in order to keep centrifugal accelerations as small as possible (Figure 1A,B). Consequently, the diameter of the containers is kept in the range of a few mm. The underlying principle becomes obvious by rotating small particles, which are forced on circular paths. The radius of the circular paths depends on the rotational speed and the density difference between the objects and the surrounding liquid [24,26]. The speed has to be optimized between “too slow”, resulting in sedimentation, and “too fast”, resulting in centrifugation. At appropriate speed, unilateral movement and thus the sedimentation of particles is prevented. By transferring this principle on the cellular level and the current thinking that gravity is perceived by tension and pressure in the interaction between cytoskeletal elements and mechanosensitive ion channels [21], we can approach a speed without unidirectional stimulus on these structures. Under this condition, the exposed systems “feel” like microgravity. Centrifugal forces of the order of 0.008 *g* to 0.018 *g* are generated with a diameter of max. 4 mm during a rotation speed of 60 rpm [27].

Depending on the scientific questions, a broad portfolio of clinostat-types has been developed, for the cultivation of adherent cells or cells in suspension, online measurement of kinetic responses by combination with live cell imaging [23].

In the case that a second axis of rotation is added, this kind of device is called 3D clinostat or Random Positioning Machine (RPM) (Figure 1C), depending on the mode of operation. In principle, a 3D clinostat and an RPM have the same mechanical design, but they differ fundamentally in the operation mode of their rotation axes. The 3D clinostat rotates with constant direction and speed. In the case that the two axes are rotated independently at random speeds and with random directions according to a sophisticated computer program, this design is called Random Positioning Machine (RPM). The principle is to average the impact of gravity and approach its influence to zero over time, an assumption that depends on the sensitivity of the exposed system [28,29]. Experiments with highly sensitive reporter systems for shear forces, however, showed a significantly different response when exposed on an RPM (real random and real direction mode) compared to their exposure on a fast-rotating 2D clinostat, indicating significantly higher shear stress on the RPM [30]. In another comparative approach, the team of Grimm et al. demonstrated comparable morphological structures (organoids) and comparable cellular signaling pathways changes, but also differences [31]. As an example, exposure of human follicular thyroid cancer cells (FTC-133) cultivated either on the 2D clinostat or the RPM revealed spheroid formation on both devices, but a different expression of selected genes or secretion of a considerable number of cytokines [32].

Another rotation-based simulation platform is the Rotating Wall Vessel (RWV, also called Rotating Cell Culture Systems RCCS, initially developed by NASA), consisting of a relatively large cylinder originally in the range of up to 20 cm diameter continuously rotating perpendicular to the Earthly gravity vector (Figure 1D). The RVW is used to examine cell-cell interactions and underlying signal cascades to elucidate the basics of tissue formation. Comparative studies, e.g., done by Hammond et al. [33], revealed differences in the responses of yeast in the RWV versus RPM with respect to gene expression changes mediated by shear stress promoters, redox status, and apoptosis. The authors concluded that the forces induced by random positioning and constant RWV rotation are different and distinct. This example also indicates that induced shear stresses at the membrane surface are likely to be important variables affecting how cells sense gravity [34,35].

As with most instrumentation, prior experience with this kind of bioreactor design has been used to improve the system. Next-generation bioreactors aim at producing organoids at reduced volumes, with the prevention of significant failure modes of RWV, e.g., by decreasing fluid shear perturbations associated with media perfusion and bubble formation [36].

This overview demonstrates the necessity to verify results from ground-based simulation approaches to those obtained in r-µ*g* conditions to avoid misinterpretations, to learn and understand device-specific characteristics and choose the appropriate simulation approach. Control experiments concerning accelerations, vibrations, and shear stress are necessary to exclude a potential impact of non-gravitational side effects. In any case, alterations of the constant influence of gravity bear experimental challenges to increase our knowledge in cellular physiology and biotechnological applications such as tissue engineering and, finally, the optimal preparation for space experiments under r-µ*g* conditions.

### 2.2. Real Microgravity Research Platforms

The force of gravity, which gives rise to body weight, is a relatively constant parameter on Earth. Due to the mass of Earth (~6 × 10^24^ kg), objects are accelerated towards the center of the planet with a gravitational force of 9.81 m/s^2^. In order to eliminate the gravitational pull of Earth, we would have to travel very far. However, a weightless environment can be created by dropping objects in a vacuum. The orbiting International Space Station (ISS) can also be regarded as in a state of free fall. However, due to the high horizontal velocity, the station’s flight trajectory never touches the Earth’s surface.

#### 2.2.1. Drop Tower

The ZARM Drop Tower in Bremen is the best-known facility in Europe and regularly used to conduct r-µ*g* experiments (for further information, go to: https://www.zarm.uni-bremen.de/en/drop-tower/general-information/how-does-the-drop-tower-work.html, accessed on 1 February 2022).

Inside the drop tower, a large experiment container is lifted to a height of about 120 m, resulting in almost 5 s of µ*g* when dropped in the vacuum. In catapult mode, however, this container is accelerated from the ground to reach the top of the tower before falling back. This procedure doubles the total µ*g* period to almost 10 s. Besides the relatively short time available to expose experiments to weightlessness, the quality of microgravity reached by using the drop tower facility is excellent (10^−6^ *g*) and even better than at the ISS [37].

#### 2.2.2. Parabolic Flight Maneuvers

Parabolic flight maneuvers facilitate a real weightless environment by compensating for all the forces acting on an object inside an airplane. During such a maneuver, the aircraft is flying on a Keplerian trajectory [38]. Creating a weightless environment by flying a parabolic trajectory on an airplane is demanding because the pilot must simultaneously account for the magnitude of several forces. For instance, once the plane has reached cruising altitude and accelerated to cruise speed, the pilot must ensure that thrust is provided to overcome the total drag of the airplane only. Furthermore, the pilot must control the airplane’s pitch so that the flight path follows a free-fall ballistic Keplerian trajectory to generate a centrifugal force that counterbalances gravity precisely. In addition, the width of the parabolic arch flown depends on the airplane’s speed, and thus, the pilot must be aware of that. During a campaign, there are typically three flights with around 31 parabolas per flight. For each parabola, there are also two periods of increased gravity (~1.5–1.8 *g*), which last for 20 s prior to and following the 20 s of µ*g* in a range of ~10^−2^ *g*. These acceleration profiles and corresponding changes have to be considered to interpret the results. Control experiments with respect to the impact on vibration and hypergravity must be performed, and data needs to be discussed in this context [39,40].

**Figure 2:**
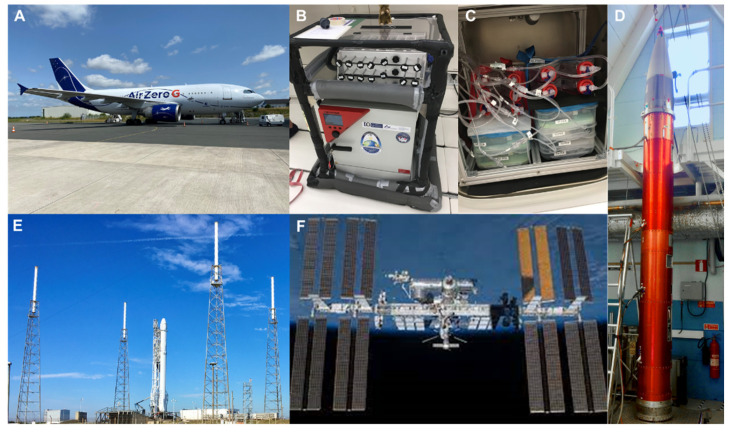
Real microgravity platforms. (**A**): 37th DLR Parabolic flight campaign, Airbus A310 Zero-*g* aircraft from the company Novespace in Paderborn, Lippstadt Airport, on 18 July 2021, (**B**): the PFC flight rack with an incubator and (**C**): open door of the incubator and boxes containing the cell culture flasks with DU-145 PCC. (**D**): Payload of a TEXUS-type sounding rocket (SSC, ESRANGE, Kiruna, Sweden; (**E**): SpaceX CRS-8 rocket on the launch pad, Kennedy Space Center (KFC), FL, USA; (**F**): the International Space Station.

Creating a weightless environment through parabolic flight maneuvers can be done by any aircraft, from gliders [41] to military fighter jet aircrafts [42]. However, the faster the plane flies, the longer the duration of the µ*g* period generated. This period ranges from a little bit longer than 5 s in a glider to almost 60 s when flown by the fighter jet aircraft Northrop F-5E. The major limiting factor, however, is the available payload space. Larger transportation aircraft thus provide an ideal compromise between the µ*g* period generated and the space available. Space agencies like NASA or ESA use larger airplanes to accommodate bigger experiment hardware that needs to be powered separately (Figure 2A–C). ESA engages the company Novespace to carry out parabolic flight campaigns on board a specially adapted Airbus A310 aircraft (for more details on ESA parabolic flight campaigns, go to: https://www.esa.int/Science_Exploration/Human_and_Robotic_Exploration/Research/Parabolic_flights, accessed on 11 February 2022).

#### 2.2.3. Sounding Rockets

Experimental payload mounted on sounding rockets (Figure 2D) can also be exposed to r-µ*g*. Such rockets have been used for scientific purposes since the late 1950s; at that time, the main objectives were meteorological observations and upper atmosphere studies. Sounding rockets provide a high quality of µ*g* in the range of 10^−4^ to 10^−6^ *g* for several minutes. However, during launch, the samples experience acceleration up to 8 *g* for a very brief moment, which needs to be taken into consideration, especially when investigating live samples. Depending on the rocket design, the microgravity phase has a range of minutes. After this period, samples are exposed to high *g*-loads during re-entry (though only for a very few seconds).

Some experiments are performed in parallel on an onboard 1*g*-centrifuge to discriminate between the real µ*g*-effects on the cells and accelerations and vibrations during launch and landing.

#### 2.2.4. International Space Station

The ISS (Figure 2F) laboratories have opened the ultimate access to r-µ*g* so far. In general, the ISS provides µ*g*-values around 10^−5^ *g*, periodically increasing to 10^−4^ *g* during a re-boost phase that happens around once per month. Through this action, which lasts no longer than 30s, the ISS is adjusting its orbit. Several facilities are available on the station to allow the conduction of biological experiments. The Europeans maintain the “BIOLAB,” located in the European “Columbus” Laboratory, and the stand-alone facility “KUBIK.” These setups provide ideal environments for numerous biological experiments, e.g., on bacteria, fungi, plants, and mammalian cells. “KUBIK” is a relatively simple facility that provides 0–2 *g* (in 0.2 *g* steps) and temperatures between 6 °C and 38 °C. A variety of available experiment hardware containers fit these facilities, which stem now from 18 years of operation. Likewise, many different experiment containers are available for the “BIOLAB” depending on the scientific demands. Besides µ*g*, the “BIOLAB” provides a centrifuge for generating 10^−3^ to 2 *g* in a controlled environment (18 °C to 40 °C, gas exchange, and the regulation of the humidity from 60–90%). Furthermore, the “BIOLAB” offers direct manipulations of the experiment by ground commanding [43].

## 3. Definition of Cancer and Cancer Stem Cells

### 3.1. Definition of Cancer

Cancer is the second leading cause of death in the Western world, only surpassed by cardiovascular diseases [44], and similar trends are observed in developing countries with modern urban lifestyles. The name cancer originates from the Greek word “karkinoma,” meaning limbs of a crab, which describes the expanded veins that spread out from some breast tumors. Later, this term was translated to cancer in Latin (and Krebs in German). In a simplified view, cancer can be defined as a collection of more than 100 diseases that progress over time and that share the common feature of uncontrolled cell growth. This results in a mass of cells termed a neoplasm (from Greek, new formation) or tumor. Hence, cancer originates when a cell overrides the well-controlled restraints of proliferation and starts to follow its own trajectory of cell division [45]. Cancer is therefore described as a proliferative, invasive, and metastatic disease triggered by an accumulation of genetic aberrations—either in the chromosomes and/or in the mitochondrial DNA—that randomly generate a malignant cell [46,47]. These aberrations result in the deregulation of pathways that introduce the possession of self-sufficient growth signals, insensitivity to anti-growth signals, infinite replication capacity, avoidance of apoptosis, persistent angiogenesis, and the ability to invade other tissues [48]. Even though the non-coding space of our genome is more than 50 times bigger than the coding exome, whole-genome analysis has suggested that non-coding driver mutations in human cancer are relatively infrequent [49].

Cancer can develop in almost all tissues of the human body, and the essential processes leading to cancer share several elements in all forms of the diseases. Another similarity is that cancer initiation may be propelled by transformed differentiated cells or tissue-resident stem cells [46]. The genetic aberrations leading to cancer development can be induced by cellular processes, such as chronic inflammation, genetic predispositions, or environmental factors, including carcinogens or exposure to radiation [50]. Cancer is a multistep process, meaning that a series of alterations and successive clonal expansions result in tumor formation across time. An essential observation is the rise in incidences with age. In most cases, cancer is an acquired disease requiring years to occur before each of the accidental changes have exerted their effect [45]. However, an important lesson from our understanding of cancer is that each form of the disease has its distinctive features [51].

### 3.2. Cancer Stem Cells

Research across several recent decades has revealed that tumors are comprised of various distinct populations of cancer cells that are important for tumor heterogeneity [52]. Despite only populating a small part of tumors, stem-like progenitor cells, also known as tumor-initiating cells (TICs) or cancer stem cells (CSC), were recently found to be key drivers of cancer initiation and progression [52]. However, the relationship between cancer and stem cells was demonstrated as early as 1964 [53]. Similar to normal stem cells, CSCs also have the capacity of self-renewal and the ability to differentiate. Importantly, CSCs lack the essential control system to avoid uncontrolled proliferation [54], and due to the high resistance to current therapeutic approaches, the presence of these cells is a major driver in the failure of cancer treatment [45].

CSCs may derive from normal cells, or stem cell mutations commenced by chronic inflammation, alterations in the environmental milieu, or by epithelial-to-mesenchymal transformation (EMT) [55,56]. Hence, stem cells can mutate directly into CSCs, whereas epithelial cells require a two-step process to become a CSC. The latter involves an initial phase converting the cell into a cancer cell, followed by EMT [52]. Other studies have also pinpointed cell fusion and metabolic reprogramming of CSCs as important steps in the origin of CSC [51]. A particular population of CSCs is constituted by mesenchymal stem cells (MSCs) that differentiate into cells of mesodermal characteristics, and the interplay between MSCs, CSCs, and cancer cells is an interesting topic for a better understanding of the initiation and progression of cancer [52].

The presence of CSCs has been recognized in various solid tumors using shared surface markers of different cancer types. These include epithelial-specific antigen (ESA), aldehyde dehydrogenase (ALDH), CD15, CD20, CD24, CD29, CD34, CD44, CD49, CD90, CD133, and CD271 [45,51]. Another CSC characteristic is the plasticity enabling the transformation between stem cells to non-stem cells and vice versa. Plasticity leads to the development of heterogeneous populations [57], and EMT is perhaps the most important process in CSC plasticity [51]. Based on these and other findings, it has therefore been suggested that phenotypic plasticity is a new paradigm for better understanding tumor initiation and progression [58]. Undoubtedly, CSCs should be considered a prime therapeutic target for cancer treatment.

## 4. Cancer Research in Microgravity

### 4.1. CSC Exposed to Microgravity

As outlined in the previous section, tumor cell heterogeneity and especially the existence of CSCs in tumors is a major problem in the failure of cancer treatment. Since µ*g* delivers an exceptional environment for cell culture and has been shown to stimulate cellular changes and processes that could not be achieved under normal gravitational conditions [59], the investigation of how CSCs act under µ*g* is an interesting topic. Even though, to our knowledge, the number of published studies on CSCs exposed to µ*g* conditions is limited, researchers have initiated work in this essential field during recent years [59,60]. Most knowledge has been obtained from thyroid, breast, and prostate cancer cells studies. Under r-µ*g* in space or during s-µ*g*, these cells display several morphological changes and alternate their cultivation behavior. Notably, these cancer cells are able to differentiate into two distinct phenotypes under µ*g* conditions; one population being maintained as adherently cells located on the bottom of the cultivation flask, and another population aggregating into three-dimensional (3D), multicellular spheroids (MCS) [61,62,63,64,65,66,67]. Such µ*g*-engineered MCS may thus delineate a model of intermediate metastasis by mimicking the diverse interplay between monolayer culture and the in vivo tumor [59]. As these MCS have similar features as the primary tumor and exhibit stem-like features simultaneously, MCS of various cancer types obtained under reduced gravitational conditions may thus represent a unique model for the study of CSC biology.

The pentaspan transmembrane glycoprotein CD133 is a cellular surface marker expressed in embryonic stem cells and various CSCs. Kelly et al. have isolated CD133-positive cells from various cancer cell types and found that they grow faster under s-µ*g*. Moreover, osteosarcoma cells, typically highly resistant to conventional therapies, lose their resistance to several chemotherapeutic agents like doxorubicin and cisplatin under s-µ*g*-exposure [68]. Like Kelly et al. in osteosarcoma CSCs, Pisanu et al. noted a significant increase in apoptosis in lung cancer CSCs under s-µ*g* conditions [69]. This study is dedicated to the stemness features of H460 cells in the altered morphogenetic field under s-µ*g*. Since high ALDH activity and high *Nanog* and *Oct-4* mRNA expression were described previously as stable biomarkers for both stemness quality and spheroid forming ability [70], Pisanu et al. investigated these factors in second-generation spheroids grown from single cells, picked from first-generation spheroids. Compared to first-generation spheroids, they found a reduction of ALDH activity and a decrease in *Nanog* and *Oct-4* mRNA expression and inferred a loss of some stemness features [69]. Yeast Associated Protein (YAP), along with β-catenin, is a known regulator of cancer cell stemness. Arun et al. investigated stemness of HCT116 cells under s-µ*g* using the surface expression analysis of CD133 and CD44 in context to YAP expression and localization and resumed, in general, a CSC increase under s-µ*g* [71]. Simultaneously, they observed an increase of autophagy core complex proteins, an elevation of aneuploidy, and the presence of giant cells with multiple nuclei. In conclusion, HCT116 cells react to s-µ*g* with increased but staggered autophagic flux and increased stemness.

The following paragraphs contain a comprehensive update on the intensive research on cancer cells exposed to µg of different cancer types, including thyroid, breast, prostate, gastrointestinal, and lung cancer, as well as skin tumors.

### 4.2. Thyroid Cancer

According to the latest Global Cancer Observatory survey from 2020, thyroid carcinomas (TC) are responsible for 586,000 cancer cases worldwide [72]. The American Cancer Society recently estimated that there will be about 43,800 new cases of TC (11,860 in men and 31,940 in women) and about 2230 deaths from this type of tumor (1070 men and 1160 women) in the United States in 2022 [73].

Thyroid tumors are divided into several categories: (1) differentiated tumors, which include papillary, follicular, and Huerthle cell tumors, (2) medullary and (3) anaplastic tumors [74]. In general, patients with differentiated TC have a long-term survival rate of almost 90%. In contrast, poorly differentiated TC types show a rather discouraging long-term survival rate of under 10% due to their resistance to standard treatment options [75,76]. Treatment options for advanced and radioiodine-refractory SDCA are multikinase inhibitors, immunotherapy, and chemotherapeutic agents [75,77]. In general, the survival rate is still low, and therefore new research approaches with new technology are necessary.

For more than 20 years, thyroid cancer cells (TCC) and normal thyroid cells have been examined under s- and r-µ*g* [78,79]. With this new technology, we are trying to expand our knowledge in cancer research. Cells grown in weightlessness show changes in morphology, the cytoskeleton, cell adhesion, migration, the extracellular matrix, gene expression, and protein synthesis as well as protein secretion [79,80,81].

#### 4.2.1. Thyroid Cells and Thyroid Cancer Cells Exposed to Real Short-Term Microgravity

Normal FRTL-5 thyroid cells were exposed to microgravity during the TEXUS (TX)-44 mission (launched 7 February 2008, from Kiruna, Sweden). R-µ*g* prevailed on the sounding rocket for 6 min and 19 s. In µ*g*-conditions, the cells revealed an irregular shape with condensed chromatin, and changes in the cell membrane with shedding of the TSH receptor in the culture medium, an elevation in sphingomyelin-synthase and Bax proteins [78].

ML-1 follicular thyroid cancer cells exposed to 31 parabolas of a parabolic flight (PF) showed changes in the structure and organization of the cytoskeleton as well as significant differences in the gene expression of cytoskeletal genes compared to ground controls. After the 31 parabolas of a PF, the *ACTB* and *KRT80* mRNAs were upregulated in ML-1 cells [82].

Postflight molecular biological analyses of the FTC-133 cell material after the TX53 sounding rocket mission were performed [83,84]. Pathway analyses revealed central functions of *VEGFA* and *EGF* [83]. Elevated mRNAs of the ECM genes *FN1*, *SPP1*, *TGFB1*, *TIMP1*, *MMP1*, *MMP3*, and *MMP14* were detected [84]. In addition, the cell adhesion genes *ICAM1* and *VCAM1*, the focal adhesion factors *CFL1* and *CDH1*, as well as cytokines *IL6* and *CXCL8*, were upregulated in r-μ*g* samples. All these factors have demonstrated their gravisensitivity [84] and involvement in MCS formation.

Using a compact fluorescence microscope (FLUMIAS) for fast imaging of living cells under real weightlessness during a TX mission, cytoskeletal changes in vital FTC-133 TCC that expressed the Lifeact-GFP marker protein could be demonstrated by on-board analysis [85]. This experiment confirmed earlier findings on PFA-fixed cells exposed to µ*g*.

Remarkably, the FLUMIAS microscope showed significant changes in the F-actin cytoskeleton in connection with weightlessness during the rocket flight [85]. In addition, this microscopic technology was used to visualize F-actin during the 24th DLR-PFC. The data of this experiment confirmed earlier findings on PFA-fixed cells exposed to µ*g*. Moreover, after the 31st parabola, cytoskeletal genes were differentially expressed [85].

#### 4.2.2. Thyroid Cancer Cells Cultured for a Longer Time in Space

With a suitable fully automatic flight hardware, FTC-133 cells were cultured in space. Special hardware (SimBox or CellBox-1/-2; both developed and built by Airbus, Defence & Space (ADS), Friedrichshafen, Germany) was used during three different spaceflights (SimBox, CellBox-1, and CellBox-2). The TCC was cultivated for ten days in space and analyzed postflight with cell and molecular biological methods [65,86,87,88,89,90].

The Shenzhou-8/SimBox experiment showed the formation of large 3D aggregates by the TCC, together with an altered expression of the *EGF* and *CTGF* genes under real weightlessness in space [87]. Interestingly, the spheroids formed during the spaceflight samples had a diameter of 5 to 10 mm, while the MCS engineered on the RPM, carried out in parallel to the space mission, were significantly smaller with a diameter of 2–3 mm. In addition, significant changes in the gene expression pattern could be observed in parallel to spheroid formation [88]. The microarray analysis revealed 2881 significantly regulated transcripts after the 10-day spaceflight. Genes in several biological processes, including apoptosis, cytoskeleton, cell adhesion, extracellular matrix, proliferation, stress response, migration, angiogenesis, and signal transduction, were significantly changed [88].

The CellBox-1 mission [89,90] had flown FTC-133 cells to the ISS (SPACEX CRS-3). The rocket was launched on 18 April 2014. The Dragon space capsule with the fixed ISS samples landed on 20 May 2014 in the Pacific in California, USA. However, the launch of the rocket was delayed several times at the beginning of the CellBox-1 experiment. This led to the fact that the cell layer (monolayer) was confluent at the time of lift-off. A confluent cell monolayer was fixed by RNAlater at the end of the experiment in space. No spheroid formation was observed during the CellBox-1 mission, but 180 proteins were identified in the cells that could be isolated from the fixed monolayers [90].

The unexpected finding was that the FTC-133 thyroid cancer cells did not form spheroids during their flight to the ISS or during their stay on the ISS, which was very likely due to the delay in take-off, which forced the cells to pre-incubate for a longer time. The cells grown in monolayers, the high cell density of which prevented spheroid formation during space travel, had an increased content of proteins involved in the regulation and composition of the extracellular matrix (ECM). It, therefore, seems very likely that a firm anchoring of FTC-133 cells to ECM proteins of a confluent cell monolayer could prevent the spheroid formation and is accompanied by phosphorylation of profilin-1 [90].

The culture supernatant of the cells was collected in a special container next to the incubation chamber of the flight hardware and stored at low temperatures until it was analyzed using Multi-Analyte Profiling (MAP) technology. The secretion of the cytokines IL-6 and IL-8 was significantly increased in the space samples compared to the ground samples (IL-6: 4.46-fold; IL-8: 3.65-fold) [89].

In addition, the vascular endothelial growth factor (VEGF) was significantly increased by 2.63 times compared to the ground samples. VEGF promotes neoangiogenesis and tumor growth and is, therefore, an important candidate for tumor therapy [75]. Interestingly, the level of the VEGF secretion during the Shenzhou-8 spaceflight was ten times higher than the level of the CellBox-1 mission. However, in Shenzhou-8, the VEGF secretion of the flight samples was lower than that of the ground samples. One explanation could be the different growth behavior of the cells [88,89].

The Cellbox-2 space mission to the ISS investigated the effect of r-µ*g* on the transcriptome and proteome of FTC-133 cells [65]. MCS were detectable in all flight samples but also an adherent 2D monolayer. In general, exposure of the cells to space conditions exhibited antiproliferative effects, influenced growth behavior, and altered the gene expression of a large number of genes involved in growth, adhesion, angiogenesis, and metastasis. Remarkably, the FTC-133 cells showed a suppression of NF-κB and ERK signaling in response to r-µ*g*. In particular, the results provided indications that gravity influences the expression of cytokines, the expression of genes of the focal adhesion complex, and the ECM [65].

#### 4.2.3. Thyroid Cancer Cells and Simulated Microgravity

TCC have been exposed to the different ESA-ground based facilities like the RPM and the 2D and the 3D clinostat (Figure 1).

S-µ*g* induced various changes in TCC exposed to the RPM, such as early changes in the cytoskeleton, ECM, focal adhesion molecules, proliferation, the rate of apoptosis, migration, and growth [31,32,61,79,91,92,93,94,95]. The major finding was the formation of MCS. The investigated TCC and cells of other tumors (e.g., breast carcinoma, prostate carcinoma) grew in r- and s-µ*g* after different exposure times in the form of 3D MCS [16,17,20,21,22,23,24,25,26,27,28,31,32,61,65,87,88,91,92,93,94,95]. Some spheroids show a great resemblance to metastases, and some have a compact structure like 24-h MCF-7 breast cancer cells or three-day PC-3 MCS, whereas five-day MCF-7 MCS exhibit a glandular shape [62,63].

Compared to 1*g*-MCS, which were generated using ‘hanging drop,’ ‘liquid overlay,’ or the ‘spinner flask’ technique, the FTC-133 spheroids generated under µ*g* were characterized by a lack of necrosis in the center of the MCS [61].

These 3D constructs are therefore particularly suitable as a model of metastasis and could also be used in the future for pharmacological tests or co-culture experiments (angiogenesis models), for example, to reduce the number of animal experiments. A first approach was done using dexamethasone, which inhibited the dose-dependently spheroid formation of FTC-133 cells exposed to the RPM [96].

More information with respect to target research using microgravity conditions was obtained by the application of omics and semantic analyses [97,98,99].

The cells growing in the form of an MCS not only show a different phenotype but also behave differently than those growing in flat monolayers under normal gravity, and they mimic conditions in the human body more closely. Therefore, the MCS has become an invaluable model for studying metastases and developing new strategies for cancer treatment through drug targeting. Weightlessness deeply affects processes such as apoptosis and induces structural changes in the cytoskeleton and ECM that are known to affect cell growth [79]. Findings from µ*g*-research could be used to complement conventional cancer research and to localize the cellular changes that lead to carcinogenesis and promote progression and metastasis. This knowledge, in turn, could lead to novel therapies that could improve patients’ quality of life or potentially contribute to new preventive countermeasures.

Healthy thyroid cells can also be cultivated on the RPM for several days under s-µ*g* [61,80]. Normal thyrocytes formed MCS within 24 h. Cytokines seem to be involved in the initiation of MCS formation via focal adhesion proteins [80]. Furthermore, normal thyrocytes can be used for tissue engineering [100]. Artificial human thyroid organoids have been generated in a rotating cell culture system in the presence of keratinocyte growth factors. These constructs structurally resembled natural thyroid tissue [100]. This report showed that the clinorotation of normal thyroid cells supports the 3D formation of thyroid cells, a finding which was also observed when TCC were cultured on a 2D fast-rotating clinostat [32].

Research in space medicine and gravitational biology today makes it possible to find new proteins in different cells and changes in protein synthesis and secretion. Altered gravitational conditions provide new technologies that help detect changes in proteins that may become new targets for tumor drug development. Further μ*g* studies should be performed to investigate tumor growth and regulation. We recently examined changes in SOX transcription factors in FTC-133 follicular cells exposed to s- and r-µ*g* and determined the mRNA expression of *SOX9* and *SOX11* in adherently growing and MCS cells [74]. The SOX family is involved in the progression and metastasis of carcinomas [74]. Future studies addressing the role of SOX family members, particularly the SoxF group, in radioiodine-resistant differentiated thyroid tumor cells are of great interest.

Recent results from cancer research in µ*g* have taught us that focal adhesion molecules play an important role in inhibiting proliferation and metastasis in melanoma cells [101]. Therefore, more intensive TC research should be carried out focusing on focal adhesions in µ*g*, using, e.g., live-cell imaging with the FLUMIAS microscope [85,102] on board the ISS or on sounding rockets. µ*g*-research as part of cancer research could provide a significant step towards better treatments. A few years ago, various proteins in the thyroid tissue were detected for the first time with the help of s-µ*g* [103]. Some of them are currently being investigated in pharmacological in vitro tests. Table 1 summarizes the results discussed above.

### 4.3. Breast Cancer

Breast cancer (BC) is still the most common cancer in women and accounts for 30% of female cancer worldwide. The ratio of BC to death is 15% [107]. The latest Global Cancer Observatory survey (GLOBOCAN) in 2020 estimated 2.3 million new cases of female BC and 684,996 death incidents worldwide [72]. BC has a heterogeneous nature and is broadly classified into three types: Luminal (Luminal A, the most common type, and Luminal B), HER2+ (the rarest), and triple negative breast cancer (TNBC). All these types of cancer differ in their prognosis, therapeutic response, disease progression, and organs of metastasis.

Although many drugs are available for BC treatment and approved by the FDA in the USA [108], the adverse effects of the available drugs and the development of drug resistance due to prolonged application of these drugs make it necessary to find alternative strategies and new drugs for BC treatment. Additionally, µg can be a complementary tool for future cancer treatment. It may open a new window for clinical cancer therapies as BC cells (BCC) behave differently under r- and s-µ*g* and produce 3D multicellular spheroids similar to microtumors in vivo, which deliver new information about novel targets necessary for the process of new drug development and drug testing.

Like other cancer cell types, BCC are also affected by r- and s-µ*g* conditions. Previous studies have shown that µ*g* affects the cell invasion, adhesion, migration, cell cycle, vinculin expression, and apoptosis in BCC [109].

#### 4.3.1. Breast Cancer Cells Exposed to Real Microgravity

Nassef et al. [110] reported in 2019 that MCF-7 BCC exposed to r-µ*g* for brief periods (TEXUS sounding rocket for 6 min and parabolic flight maneuvers for 22 s) showed an enhanced gene expression of *KRT8, RDX*, *TIMP1, CXCL8,* and a reduced *VCL* gene expression along with a decreased E-cadherin protein synthesis. Live-cell imaging demonstrated major rearrangements of the cytoskeleton with alterations of F-actin and alpha-tubulin with holes. These findings were accompanied by the appearance of lamellipodia-like (LP) and filopodia-like (FP) structures in the F-actin cytoskeleton [110].

The MDA-MB-231 triple-negative breast cancer cells (TNBC) investigated during parabolic flight maneuvers showed an early upregulation of *ICAM1*, *CD44*, and *ERK1* mRNAs and a delayed upregulation of NFKB1, NFKB p-65, and annexin-A2 protein [111].

Vassy et al. [112] demonstrated that MCF-7 cells flown in a photon capsule in space for time periods of 1.5, 22, and 48 h showed a lower signal transduction (phosphotyrosine), altered microtubules, and chromatin structure. The authors also reported prolonged mitosis and reduced cell proliferation in space-flown BCC compared to ground control cells [112].

#### 4.3.2. Breast Cancer Cells and Simulated Microgravity

When BCC (MCF-7 and MDA-MB-231 cells) were exposed to an RPM, the BCC transformed from a 2D monolayer into 3D spheroids. The RPM-exposed cells revealed alterations in cytoskeletal proteins, changes in ECM components, and FA factors. A 24-h RPM exposure induced a significant down-regulation of *ITGB* and *LAMA3* mRNAs in AD cells and MCS of the MDA-MB-231 cell line. The *VCL* mRNA was significantly reduced in MDA-MB-231 MCS cells [66]. Moreover, the *FAK1, PXN, TLN1, VCL,* and *CDH1* mRNAs were significantly down-regulated in adherent MCF-7 cells cultured for 24 h on the RPM. Furthermore, *PXN, TLN1,* and *CDH1* were down-regulated in MCS, whereas *VCL* and *LAMA3* mRNAs were not changed in MCF-7 BCC [66]. The interaction analyses indicated a central role of fibronectin, vinculin, and E-cadherin in MCS formation. Live cell imaging of β-catenin-transfected MCF-7 cells revealed a nuclear expression of β-catenin in 1*g* and RPM-AD cells. The target genes *BCL9, MYC,* and *JUN* of the Wnt/β-catenin signaling pathway were differentially expressed in RPM-exposed MCF-7 cells [66]. Together, vinculin and β-catenin are involved in forming 3D spheroids during a 24-h RPM exposure.

Similar data were obtained when MCF-7 cells were cultured on a clinostat for 24, 48, and 72 h [109]. The results showed an effect on BCC invasion, migration, adhesion, cell cycle, cell apoptosis, and vinculin expression by the clinostat-exposure.

MCF-7 BCC exposed to the RPM showed a down-regulation of *VEGFA*, *FLK1*, *CASP9*, *CASP3,* and *PRKCA* mRNAs, interfering with 3D cell aggregation [62].

A further study using Gene Array Technology showed significant upregulations of *ANXA1*, *ANXA2*, *CTGF*, *CAV2*, *ICAM1*, *FAS*, *CASP8*, *BAX*, *TP53*, *CYC1*, and *PARP1* in MCS compared with the 1*g* control and AD cells [113]. Furthermore, RPM-exposed MCF-7 BCC incubated for 24 h with the NFκB inhibitor dexamethasone showed a dose-dependent inhibition of spheroid formation. The glucocorticoid dexamethasone functionally inhibits NFκB and the NFκB-dependent gene expression suggesting that NFκB plays a crucial role in spheroid formation in tumors [113].

MCF-7 cultured for 14 d on the RPM formed 3D MCS and grew in parallel as an adherent monolayer under s-µ*g* conditions. Subsequent proteome analysis revealed that the cell junction protein E-cadherin was diminished in MCS cells, whereas the E-cadherin autodegradation pathway proteins were enhanced and c-Src (proto-oncogene tyrosine-protein kinase c-Src) was detected. MCS formation was prevented by inhibiting c-Src but promoted by antibodies blocking E-cadherin activity [114].

The BCC line CRL-2351 exposed to an iRPM for 24 h showed the formation of 3D spheroids [115]. *BRCA1* and *VCAM1* were elevated in RPM-AD cells compared to 1*g*. *VIM* was down-regulated in AD cells and MCS. No changes were found for *MAPK1*, *MMP13, PTEN*, and *TP53* mRNAs [115]. These BCC were also investigated in a long-term iRPM experiment. A distinct MCS formation together with a rearrangement of the cytoskeleton was observed. *VIM*, *RHOA*, *MAPK1*, and *BRCA1* mRNAs were significantly up-regulated in AD RPM cells and MCS. *ERBB2* showed a significant increase only in MCS, whereas *RAB27A* and *VEGFA* showed no significant changes in the gene expression [116].

An earlier study showed that 48-h RPM exposure alters the cytofluorimetric profile and slows down fundamental metabolic activities (glucose uptake, methionine uptake/incorporation, and thymidine incorporation) in BCC [117]. S-µ*g* did not significantly affect the expression of most proteins related to the cell cycle and apoptosis, whereas it altered the expression of stress proteins.

The 3D growth and detection of different phenotypes of BCC exposed to s-µ*g* were also confirmed by other groups. MDA-MB-231 BCC exposed to s-µ*g* grew in the form of two phenotypes, adherent monolayers, and 3D aggregates. These morphological differences are mirrored by the concomitant dramatic functional changes in cell processes (proliferation and apoptosis) and signaling pathways (ERK, AKT, and Survivin). Furthermore, the cytoskeleton undergoes a dramatic reorganization, eventually leading to a very different configuration between the two populations [64]. This data supports the findings of other researchers and the results of other cell types [61,63,91,118].

Normal breast MCF-10 cells and MCF-7 BCC exhibit different counteractive mechanisms to ensure their survival on the RPM. In normal breast cells, the ERK survival pathway was activated, whereas the Akt pathway was activated in MCF-7 BCC when exposed to microgravity [119].

Bauer et al. performed a semantic study on MCF-7 BCC regarding posttranslational modifications of proteins in cells exposed to the RPM. They found that s-µ*g* influences the expression of adhesion proteins and enzymes involved in the sialylation of the extracellular domain of adhesion proteins [120].

Furthermore, the NASA developed rotating wall vessel (RWV) bioreactor was applied to engineer 3D spheroids [121]. Vamvakidou et al. [122] generated a co-culture-based heterogeneous 3D tumor model from MDA-MB-231, MCF-7, and ZR-751 cells in the RWV. The most important outcome was the temporal-spatial organization of the MCS, having central necrotic areas with higher levels of cell proliferation at the MCS periphery. These MCS can also be used as in vitro models for drug testing.

In a recent study, MCF-7 BCC were cultured in an RWV. The expressions of integrin β1, paxillin, and E-cadherin under s-µ*g* were down-regulated compared to that of the control group showing a time-dependent pattern of the decreasing EMT transcription factors snail, twist, and ZEB1 were up-regulated under s-µ*g* conditions [123]. In summary, MCF-7 cells respond to stress changes to regulate the expressions of adhesion proteins and adapt their adhesion state to the altered mechanical environment. The altered cell adhesion in response to the mechanical stress may involve the changed expression of EMT-inducing factors, snail, twist, and ZEB1 under s-µ*g* [123].

Finally, MDA-MB-231 TNBC cells exposed for seven days to the RCCS showed significantly increased lysosomal vesicles but a reduced cell migration behavior in the s-μ*g* environment compared to the 1*g* conditions [124]. The expression of BCL-2 and MMP9 decreased, whereas the expression of cyclin D3 increased in s-μ*g*.

In addition, when culturing MCF-7 and MDA-MB-231 BCC on the RCSS for five days, the cyclin D1 expression was reduced [125].

A recent study demonstrated the dysregulation of extracellular vesicles (EV) deriving from MDA-MB231 cells cultured on the Gravite device, another type of RCCS [126]. The authors demonstrated that the EV release rate decreased, but the average size increased. Proteomic studies of EV and BCC further revealed a significant correlation with GTPases and proliferation of MDA-MB-231 cells in µ*g* [126].

This chapter summarizes the effects of r- and s-µ*g* on different types of BCC. It provides a comprehensive report of many aspects of BC research under µ*g*-conditions. The µ*g*-environment altered biological processes like survival and apoptosis, proliferation, invasion, migration, and adhesion (Table 2). Thus, it seems to be a potential field to explore for BC treatment and to help establish the therapeutic links between µ*g* and novel BC treatment.

### 4.4. Prostate Cancer

In 2020, the GLOBOCAN (Global Cancer Observatory) published that prostate cancer (PC) comprises an incidence of almost 1.4 million new cases and 375,000 deaths worldwide. PC is the second most frequent cancer and the fifth leading cause of cancer death among men [72]. The 5-year survival rate with local or regional PC is high and about 100%. However, after the diagnosis of metastasis, the 5-year survival rate decreased to 31% [128]. Therefore, it is important to increase the knowledge of PC biology, genomics, proteomics, and advanced profiling technologies and establish new techniques to discover new drug development targets.

Using the High Aspect Ratio Vessel (HARV) developed by NASA a few years earlier [129], Clejan et al. had exposed human DU145 prostate carcinoma cells (PCC) to s-µ*g* conditions in 1996. They observed 3D growth of the cultures with cytodex-3 microcarrier beads with concomitant development of filopodia and microvilli-like structures. Compared to the 1*g* controls, this development was accompanied by decreased growth and increased staining of the cytoskeletal proteins cytokeratins 8 and 18, actin, and vimentin within the two-week experiment. This was particularly evident compared to the static controls, with the spinner flask control occupying an intermediate status. The elevated biomarker concentration, decreased growth, and increased differentiation of s-µ*g* DU145 prostate carcinoma cultures were interpreted by the authors as a sign of decreased aggressiveness [130]. The following year, the group published results of a 17-day DU145 PCC HARV to a transwell comparison study [131]. The s-µ*g* to 1*g* ratio of EGF (0.15), EGF receptor (0.52), TGF-β1 (1.2), TGF-β receptor (0.17), collagen IV (3.7), and laminin (2.4) was determined. The authors interpreted the composition of the selected regulatory and matrix proteins as further evidence of the reduction in the aggressiveness of the PCC cultures under s-µ*g* conditions. In 2001, Clejan et al. examined temporary effects of 15-day HARV-cultivated DU145 PCC spheroids on the signal transduction-second messenger pathways [132]. Radioassays such as HPLC and TLC have been used to determine early signal transduction pathways of lipid second messengers like, among others, DAG, ceramide, or cAMP. They observed a spheroid-specific cross-talk between the different second messengers starting with an early DAG increase in the HARV-cultivated cells.

In contrast, the microcarrier beads-supported HARV experiment of Zhau et al. under the leadership of Leland Chung focused on the androgen-sensitive human prostate adenocarcinoma LNCaP PCC in co-culture with prostate fibroblasts [133]. The co-culture was selected to establish an in vitro model for the study of normal and neoplastic prostate development and to examine molecular mechanisms of cellular signaling in response to androgen stimulation. The group observed a higher PSA expression in the co-cultured LNCaP cells. In summary, it was found that under s-µ*g*, DHT stimulated differentiation of LNCaP only occurred in co-culture with prostate fibroblasts. After a three-year delay, Chung’s group was able to perform a space shuttle Columbia STS-107 mission with PCC samples under r-µ*g* conditions in 2003 [134]. In the space shuttle mission, despite the short r-µ*g* exposure, larger organoids could be obtained than in the HARV experiment. Unfortunately, due to the tragic Space Shuttle crash in which all seven astronauts died; a major part of the data was lost.

In 1997, Ingram et al. developed a system based on HARV s-µ*g,* among others, for PC-3 PCC without microcarrier beads [135]. Compared to DU145 PCC, but particularly LNCaP, the adenocarcinoma-derived PC-3 exhibits a significantly higher metastatic potential. Ingram et al. observed upregulation of the cell adhesion molecules CD44 and E-cadherin in the 3D spheroid structures in general. Whereas no collagen IV expression was detected in PC-3 cells, the epithelial marker cytokeratin-VIII was upregulated. In contrast to the compact DU145 PC spheroids, loose cell assemblies were observed in PC-3 spheroids. Fibroblast spheroids showed increased tenascin expression when co-cultured with PC-3 PCC. The cell elongation in the spheroid outer layer and the formation of pseudo-capsules, typical for fibroblast spheroids, could not be observed in PC-3 PCC.

The results of a detailed transcriptional and immunohistochemical analysis of PC-3 spheroid development was presented in 2020 by Hybel et al. [63]. The MCS and adherent remaining cells (AD) formed by s-µ*g* in the RPM were compared after three and five days of s-µ*g*-exposure with each other and the 1*g* control. Extensive qPCR analysis of 26 genes revealed, among others, MCS to AD expression depletion of four genes *(VEGFA, AKT1, MTOR,* and *COL1A1*) after three days of RPM exposure, depletion of ten genes (*VEGFA, AKT1, MTOR, RAF1, SRC1, ERK2, TUBB, ACTB, LAMB2,* and *LCN2*) and elevation of five genes (*FLK1, COL5A5, CDH1, LAMA3,* and *FN1*) after five days of RPM-exposure. Simultaneously, a significant decrease in VEGFA and NGAL secretion rate due to s-µ*g* influence was observed. An overview of all results is presented in Table 3.

In summary, the gap of µ*g*-related PC studies between 2003 and 2022 stands out. The modern analysis capabilities of our day have been exploited to only a limited extent with respect to this disease. There is hope that more studies will follow in the future, especially studies under r-µ*g*.

### 4.5. Cancers of the Gastrointestinal System

Gastrointestinal tumor cells have been one of the first cell types used in µ*g*-research. This chapter comprises cell types from the gastrointestinal tube and organs like the liver and pancreas. Unfortunately, no gastrointestinal cancer cell types have been used in r-µ*g* research on the ISS, unless for a direct comparison between the NASA-designed RWV on Earth and an r-µ*g* experiment in 2000. The noteworthy point is that multiple co-culture experiments have been conducted for this cancer group, acknowledging the importance of cancer cell interaction with the typical cellular environment.

#### 4.5.1. Colorectal Cancer

Colorectal cancer (CRC) comprises colon cancer or rectal cancer and is the development of cancer from the colon or rectum (parts of the large intestine). CRC is the second most common cause of cancer death in the United States. In 2020, approximately 147,950 individuals were estimated to be diagnosed with CRC and 53,200 to die from the disease, including 17,930 cases and 3640 deaths in individuals aged younger than 50 years [136].

The first cell line of the gastrointestinal system cultivated in µ*g*-research dates to 1992, with the colon carcinoma cell line HT-29 [137]. The cells were grown in an RWV co-culture with fibroblasts and formed MCS with glandular structures, apical and internal glandular microvilli, sinusoid development, internalized mucin, and structural organization akin to normal colon crypt development. The results suggested that the RWV could be a new model for investigating and isolating regulatory and structural processes within neoplastic and normal tissue.

Then in 1994, Jessup et al. [138] found that s-µ*g*-exposure does not alter epithelial cell adhesion to the matrix and other molecules in human HT-29KM, CCL 188, KM-12c, and MIP-101 colorectal carcinoma cells. Their results suggest that s-µ*g* may also be useful for tissue engineering applications.

Three years later, the same group [139] tested a different CCR cell line, MIP-101, which produces minimal amounts of carcinoembryonic antigen (CEA) in monolayer cultures. MIP-101 cells cultivated in microcarrier beads and exposed to the RWV showed increases in CEA production compared to the 1*g* static flask culture group. Notably, the non-adherent petri-dish culture increased the CEA production in similar amounts. The authors concluded that 3D growth, independently of using a (non-adherent surface or a clinostat device) might enhance the production of molecules associated with the metastatic process. These results suggest that the mechanical stimuli regulate functional capabilities in colon cancer cells [139].

In 2000, Jessup et al. tested the hypothesis whether shear forces generated by the RWV increase apoptosis or differentiation in the colon cancer cells [140]. The MIP-101 cell line was tested in four experimental conditions: 1*g* monolayers, 3D growth in non-adherent surfaces, 3D growth in low earth orbit with the RWV, and rotating 3D growth in the RWV on the ground. Interestingly, µ*g* culture reduced apoptosis and increased the differentiation pattern. Moreover, the reduced rotation in µ*g* increased the CEA expression. The authors showed how shear stress modulates cell proliferation and apoptosis through pathways regulating the growth factor expression of EGF-R, TGF-alpha, or TGF-beta [140].

Another publication reported testing liver-based cell organoids in an RWV as a metastasis model [141]. The MCS were inoculated with HCT-116 colon carcinoma cells for modeling liver metastasis. The immunofluorescence staining revealed that in the 1*g* conditions, the cells displayed an epithelial phenotype, and in the organoids, the phenotype was mesenchymal. The cell surface marker expression suggests that the WNT pathway may be involved in these changes. Moreover, manipulating this pathway with WNT agonists and antagonists changed the sensitivity of the antiproliferative drug 5-fluoruracil (5-FU) [141].

Devarasetty et al. [142] cultured HCT116 colon carcinoma cells in an RWV with primary human hepatocytes in a co-culture experiment setting that included mesenchymal stem cells. Only in the presence of mesenchymal stem cells, large tumor foci formed rapidly inside the spheroids, which then increased in size over time, mainly by forming a stroma-like tissue surrounding the tumor foci. When tumor organoids were allowed to mature in the RWV, they became less sensitive to the chemotherapeutic drug 5-FU. This experiment demonstrates the potential of µg-devices for studying cancer progression and drug treatments [142].

In addition, three different colorectal cancer cells (DLD1, HCT116, and SW620) were grown in a Rotational Cell Culture System-High Aspect Ratio Vessel (RCCS-HARV) [143]. The cells tested in this device died through apoptosis under s-µ*g*. The gene expression showed upregulation of the tumor suppressors PTEN and FOXO3, which led to AKT downregulation and apoptosis induction. Furthermore, the clumps formed in µ*g* showed elevated hypoxia and mitochondrial membrane potential. This data suggests that 3D growth in s-µ*g* can be exploited to study the stress response in cancer cells, particularly in this case, stress to the loss of surface adherence [143]. This paper highlights the regulation of cell function and viability under s-µ*g* through PTEN/FOXO3/AKT signaling [143].

A further study compared two different 3D growth culture conditions: non-adherent well plates coated with agarose (3D culture at 1*g*) and cells rotating in the RCCS, which, as discussed earlier, exposes the cells to shear forces because of the rotation [71]. The authors focused on the role of RCCS-exposure in increasing stemness in human colorectal cancer HCT116 cells. Distinct features of cancer stem cells, including CD133/CD44 dual positive cells and migration in s-µ*g* which were not altered by autophagy induction or inhibition [71]. 3D 1*g* cultures and s-µ*g* increased autophagy, but the flux was staggered under s-µ*g*. Increased unique giant cancer cells housing complete nuclear localization of YAP were observed in RCCS cultures [71]. Furthermore, the s-µ*g* cells improved their migration compared to 1*g* and 3D cultures. Low shear forces increase migration capabilities compared to the static cell cultures, either 3D or 1*g*. Finally, the authors found an upregulated protein expression of Yamanaka factors (OCT4, Nanog, and SOX2) in the s-µ*g* group. In conclusion, the combined effect of shear forces caused by rotation and the lack of adherence to the surface seems to increase the stemness features [71].

Proteomics analyses of human colon adenocarcinoma Caco-2 cells exposed to a 2D clinostat revealed 38 and 26 proteins differently regulated by s-µ*g* after 48 and 72 h. Substantial fractions of these proteins are involved in regulation, cellular and metabolic processes, and localization. S-µ*g*-induced changes in the transcriptional regulation of the NF-kB pathway and CYP27A1 were detectable [144].

A recent paper published by Gouws et al. [145] reported the use of a 2D Clinostat to tissue engineer 3D LS180 cell mini-tumors, suitable for drug testing. The authors tested the efficacy of the South African plants *Sutherlandia frutescens* and *Xysmalobium undulatum* in comparison to the standard paclitaxel treatment. After 96 h, *S. frutescens* extract markedly decreased the soluble protein content while decreasing ATP and AK per protein content to below detectable limits after only 24 h exposure. *X. undulatum* extract also decreased the soluble protein content, cell viability, and glucose consumption. The results suggested that the two phytomedicines have the potential to become a source of new treatments against colorectal cancer.

#### 4.5.2. Hepatocellular Carcinoma Exposed to Simulated Microgravity

Hepatocellular carcinoma (HCC) or liver cancer is the fourth most common cause of cancer mortality, and its incidence is increasing. The liver is also a common metastatic site because of its anatomical relationships [146]. Cancer of the liver remains a global health challenge and its incidence is growing worldwide. It is estimated that, by 2025, >1 million individuals will be affected by liver cancer annually. HCC is the most common form and accounts for ~90% of the cases [147].

Approximately 21 years ago, Khaoustov et al. [148] tested HepG2 cells in a rotatory cell culture system. They performed a microarray analysis of genes differentially expressed in cells cultured in s-µ*g* during an early stage of 3D assembly, which revealed changes in the expression of 95 genes (overexpression of 85 and downregulation in 10) [148].

Some years later, in 2007, another study [149] used the same cell line, but this time with a more robust microarray profile in the RCCS. The authors performed a time-course experiment for identifying genes altered in the s-µ*g*-environment after 1, 3, and 4 d of s-µ*g*-culture, and they found 139 genes significantly altered in s-µ*g*. They compared their results with the research previously discussed and found some discrepancies between the gene sets found by each group [149].

Another study used the RWV bioreactor for tissue engineering of liver spheroids [150]. The cells formed spheroids up to 100 µm in diameter after 72 h in the RWV. A long-term culture allowed the MCS to reach 1 mm in diameter. The actin cytoskeleton acquired a cortical organization in the spheroids. Liver-specific functions like cytochrome P450 activity and albumin production were higher in the spheroids. When the MCS were transferred to regular flasks again, they disintegrated and lost functionality. These results demonstrated the importance of the physical environment on cellular organization and the hepatocyte process [150].

Another team of researchers established an in vitro 3D model of metastatic hepatocellular carcinoma by culturing MHCC97H cells on molecular scaffolds within a rotating wall vessel bioreactor [151]. The model mirrored many features of HCC in vivo, like the cancer cell morphology, tissue ultrastructure, production and secretion of proteins, glucose metabolism, and apoptosis. The MCS formed were then transplanted into nude mice livers, resulting in tumorigenesis and distant metastasis, illustrating the capabilities of s-µ*g*-research models in cancer biology [151].

Chen et al. [152] used the previously developed co-culture method in the RWV to test the dynamic expression patterns of proteins during the invasion process of HCC. Highly metastatic MHCC97H cells and a liver tissue fragment were co-cultured in the RWV to simulate different pathological states of HCC invasion. For example, during the invasion process, the time-course analysis showed dynamic gene alterations: *MMP2*, *MMP7*, *MMP9*, *CD44*, *SPP1*, *CXCR4*, *CXCL12*, and *CDH1*. The 529 common differential proteins related to HCC invasion clustered into 25 expression patterns. Some proteins even showed continuous upregulation during the entire invasion process (e.g., vitronectin, Met, clusterin, ICAM1, GSN) [152].

The same research group [153] compared 3D HCC spheroids with different metastatic potential and focused on candidate metastasis-associated genes. The highly metastatic MHCC97H cells and low-metastatic Hep3B cells were grown in an RWV after 15 days of culture. They found 70 upregulated genes (*VCAM1*, *IL1B*, *CD44*, *TNC*, *SPP1*, *FN1*, *MMP2*, *MMP7*) and 53 down-regulated genes (*CDH1*, *CTNND2*) in the high-metastatic spheroid. A functional classification found that adhesion molecules mediating cell-matrix interactions and matrix secretion were significantly higher in high-metastatic spheroids [153].

In 2019, HepG2 cells were cultured in the Gravite device, a 3D clinostat [154]. The data shows that the s-µ*g*-environment enhanced CDDP-induced apoptosis via p53-independent mechanisms. CDDP-induced ATM/p53 signaling increased under µg, and caspase-3 was cleaved earlier. S-µ*g* decreased the levels of expression of BAX and CDKN1A, which are targets of p53. Also, µ*g* increased the mRNA levels of PTEN, DRAM1, and PRKAA1. S-µ*g* reduced the levels of mTOR. Moreover, s-µ*g* increased the LC3-II/I ratio, suggesting autophagy activation. Finally, the cleaved caspase-3 was detected even in the s-µ*g* group with a mutant cell line constitutive expression of p53. This experiment indicates that the altered CDDP sensitivity (caused by s-µ*g*) occurs through p53-independent mechanisms [154].

A recent publication reported the development of an HCC (HepG2/C3A) cell-based 3D model for genotoxicity testing of chemicals by using a new clinostat model device [155]. The 21-day-old MCS were exposed to indirect-acting genotoxic compounds, polycyclic aromatic hydrocarbon, and heterocyclic aromatic amine at non-cytotoxic concentrations for 24 and 96 h. Both environmental pollutants significantly increased the level of DNA strand breaks assessed by the comet assay. The 21-day-old MCS showed higher basal expression of genes encoding metabolic enzymes than the 1*g*-culture. The compounds induced a specific up-regulation of genes implicated in their metabolism and deregulation of genes implicated in DNA damage and immediate-early response in the MCS [155].

This last study summarizes this section on liver tumors. We observed that this research field has developed a significant number of 3D MCS liver models with obvious focus on drug testing by taking advantage of the capabilities offered by the s-µ*g* environment for tissue engineering and cancer cell biology.

#### 4.5.3. Studies Using Gastric and Pancreatic Cancer Cells

Gastric and pancreatic cancer cells exposed to µg have attracted little attention from the space research community. Therefore, we should take the opportunity to explore these areas.

Recently, Chen et al. [156] studied HGC-27 gastric cancer cells and demonstrated that s-µ*g* affected lipid metabolism using liquid chromatography-mass spectrometry. The cells were cultured in an RCCS bioreactor, and the researchers identified 67 differentially regulated metabolites [156]. Phosphatidylethanolamine, phosphatidylcholine, arachidonic acid, and sphinganine upregulated significantly in s-µ*g*. In contrast, sphingomyelin, phosphatidyl-serine, phosphatidic acid, L-proline, creatine, pantothenic acid, oxidized glutathione, adenosine diphosphate, and adenosine triphosphate were significantly downregulated [156]. The human metabolome database compound analysis revealed that lipids and lipid-like metabolites were primarily affected in an s-µ*g*-environment [156].

Finally, human pancreatic carcinoma NOR-P1 cells and fibroblasts or minced human pancreatic carcinoma tissue were cultured in RCCS-4D in order to design a 3D carcinoma tissue model [157]. The NOR-P1 cultures subjected to the s-µ*g*-conditions showed more significant numbers of mitotic, cycling (Ki-67 positive), nuclear factor-kappa b activating cells, and a lower number of apoptotic cells compared to the static 1*g*-condition [157]. This study used a novel 3-D rotary cell culture system with four disposal vessels useful for engineering complex pancreatic carcinoma tissue [157].

In summary, the concise review of the available literature of cancer cells of the gastrointestinal tract cultured under conditions of s-µ*g* indicated the preferred use of the RWV and RCCS bioreactors and clinostat devices. These bioreactors have proven to be suitable for engineering 3D spheroids, organoids, or tumor tissues. An overview of all discussed studies is given in Table 4.

### 4.6. Lung Cancer

Lung cancer is one of the most common malignant tumors of the Western world. The age-standardized incidence of lung cancer ranges from 33.3 to 66.8 per 100,000 among males and 10.5 to 37.5 per 100,000 among females [158]. The disease is associated with a poor prognosis, having an overall mortality rate of 46.0 per 100,000 individuals per year in the United States [159]. Lung cancer cells (LCC) have been studied in several in vitro models with the intent to improve diagnostics and therapy. Among other experimental setups, s-µ*g* conditions have been applied to both mature and cancer stem cells.

Like in other tumors, CSCs have been identified for lung cancer [160]. In the lung, three different sections can be distinguished: the major airways, the bronchioles, and the respiratory bronchioles/alveoli. Despite a slow cell turnover of the lung epithelium, cell-subpopulations with self-renewing capacities have been identified [161]. For the pseudostratified epithelium-lined [162] proximal airways, including the trachea and the main bronchioli, keratin-expressing cells have been shown to possess capacities of regeneration and differentiation and are therefore regarded as stem cells in the major airways [163]. Particularly keratin (K)-5 [164] and k14-positive [163,165] cells are regarded as normal tracheal stem cells but also stem cells for squamous cell carcinomas. The middle airway is coated by columnar epithelium [162]. Already in the 1970s, it could be shown that Clara cells could dedifferentiate after injury and transform into a cell type similar to bronchiolar epithelial cells [166]. These Clara cells were initially seen as stem cells but are regarded as ‘facultative progenitor cells’ nowadays [167]. In Clara cell-depleted environments, however, two subpopulations of cells located in the neuroepithelial bodies have been identified as stem cell candidates for the middle part of the airways [168]. First, there are calcitonin gene-related peptide (CGRP)-positive pulmonary neuroendocrine cells that proliferate and form hyperplastic areas in a naphthalene injury model [169]. However, these cells do not show independent ability to repopulate cell-depleted airways. The second population consists of Clara-cell secretory protein-positive (CCSP+)-cells, which are both able to proliferate and differentiate after middle airway injuries. These cells are currently regarded as the ‘true’ middle airway stem cells [160,170]. Small cell lung carcinomas (SCLCs) mainly originate in the midlevel bronchioles [171]. Among SCLC cell lines, those positive for urokinase plasminogen activator receptor (uPAR) were found to have ‘stem cell-like’ properties, such as multi-drug resistance, high proliferation, and expression of the stem cell markers CD44 and MDR1 [172,173].

In the distal airways, the respiratory bronchioles and alveoli are lined by cuboidal epithelium [162]. The actual stem cell for the distal airways has not been identified yet, but a Sca-1+/CD45−/PECAM−/CD34+ subpopulation showed proliferation, self-renewal, and multilineage differentiation in culture, making them good candidates for the distal airway stem cell [174]. Concerning small and non-small lung cancer stem cells in general, a CD133+ subpopulation [175] showed properties of cancer stem cells when isolated. This subpopulation could be cultivated indefinitely and showed tumor formation after injection into immunocompromised mice [176].

To date, experiments with lung cancer stem cells under µ*g* are scarce. A PubMed search with the terms “lung cancer” and “microgravity” yields six original studies [69,177,178,179,180,181] and one review article [121]. The search terms “microgravity” and “lung cancer stem cells” yielded one original article [69] and one review article [121]. Both were also included in the search above.

To our knowledge, all data about LCC under µ*g* has been obtained in simulation devices on Earth. No experiments with LCC have been performed on a space station, nor in free fall experiments or parabolic flight maneuvers so far. As known from non-cancer stem cells, the effects of s-µ*g* can vary considerably between different cell types. While embryonic stem cells tend to stay undifferentiated, human mesenchymal stem cells differentiate into a cartilaginous lineage, and periodontal ligament stem cells differentiate into an osteogenic line [182]. The non-small lung cancer NSCLC cell line H460 is known to be rich in cancer stem cells [183]. Under s-µ*g* in a 3D clinostat, H460 cells showed decreased aldehyde-dehydrogenase (ALDH-) activity and decreased Oct-4 and Nanog expression, which may indicate loss of stemness abilities [69].

**Figure 3:**
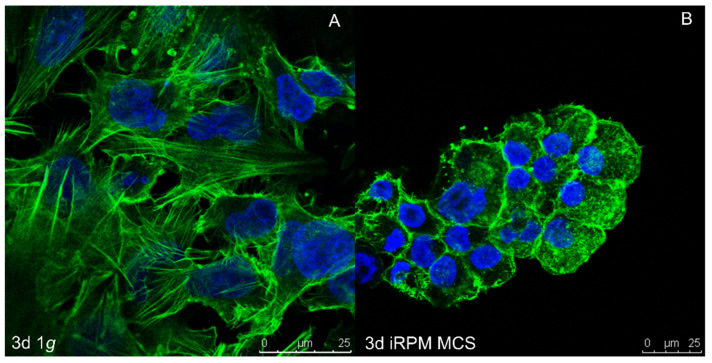
F-actin cytoskeleton: (**A**) Lung cancer cells cultured under static 1*g* conditions stained with DAPI (blue nuclei) and labeling of F-actin with phalloidin (green colored cytoskeleton) and (**B**) MCS of LCC cultured in the iRPM.

Lung adenocarcinoma cells (A549) showed no change in proliferation in a 3D clinostat, while squamous cell carcinoma cells (H1703) decreased their proliferation rate. Both cell lines showed increased migration [177]. When lung cancer non-stem cells of the cell line CRL-5889 (squamous LCC) were exposed to s-µ*g* in an incubator 3D RPM (iRPM) in our experiments, we found a significantly increased apoptosis rate, alterations in the arrangement of the cytoskeleton from longitudinal to spherical (Figure 3) and upregulation in the tumor suppressor genes *TP53*, *CDKN2A*, *PTEN*, and *RB1*, together with an increase in the corresponding gene products p14 and RB1.

Similar effects were observed by Chang et al. when human lung adenocarcinoma cells were exposed to s-µ*g* in a 2D rotating clinostat [180]. The authors reported findings in accordance with decreased metastatic potential, such as decreased matrix metalloproteinase 2 (MMP-2) activity, decreased proliferation, decreased migration, and decreased gelatinolytic activity. When human adenocarcinoma alveolar epithelial cells were exposed to s-µ*g* by Degan et al. [181], the authors also observed a decreased cell proliferation with a lower number of cells going through S phase in the proliferation cycle. Furthermore, mitochondria showed ultrastructural damage, with an altered shape of the cristae and an increased number of autophagic/autolysosomal vacuoles. Several miRNAs were changed in their expressions, but the interpretation of the significance of this phenomenon is difficult. When gene regulation of the altered miRNAs was calculated with bioinformatic tools, the 3-kinase-Akt (PIK3R1-Akt) signaling pathway was identified to be crucial in the transmission of microgravity effects. The calculated alteration of gene expression of *PTEN* and *CDKN2A/1A* was also observed in our studies [178].

In summation, s-µ*g* significantly alters the behavior of lung cancer stem cells and non-stem cells. Proliferation, stemness, and invasiveness seem to be decreased. Further identification of the underlying mechanisms may help to develop new targeted therapies. As all available data has been obtained under s-µ*g*, further experiments under r-µ*g* in space may deliver new interesting information that may be of use for metastasis studies and the development of new cancer treatments.

### 4.7. Skin Cancer

So far, all known studies of skin cancer in µ*g* have been performed on malignant melanoma, which arises from the uncontrolled proliferation of melanocytes. Although it accounts for only 1% of all skin cancers, malignant melanoma is the most lethal form and responsible for about 80% of all skin cancer deaths [184]. According to GLOBOCAN, 325,000 new cases occurred globally in 2020 (1.7%) [72]. The metastatic stage presents the greatest therapeutic challenge because it responds poorly to chemotherapy.

In 2006, Taga et al. [185] described a 50% decrease in the growth of murine B16-F10 melanoma cells, which were cultured on an RWV. An increase of melanin after 24 and 48 h indicated a differentiation. However, after the cells were inoculated subcutaneously in mice, they found a significant increase in tumorigenicity compared to conventionally cultured cells. They concluded that s-µ*g* (RWV) seems to enhance the invasive property of B16-F10 cells, although the cells showed an increase in the percentage of apoptotic cells [185]. Zhao et al. [186] later reported that s-µ*g* (RPM) promotes the apoptotic response of murine B16-BL6 cells through a combined modulation of the apoptosis (Uev1A/TICAM/TRAF/NF-κB-regulated) and DNA damage response pathways (p53/PCNA- and ATM/ATR-Chk1/2-controlled).

Further studies with B16-BL6 cells on the RPM showed reduced cell proliferation, adhesion, and, in contrast to the earlier RWV study, reduced invasiveness in vitro and decreased lung metastasis in vivo [101]. Here, the authors found down-regulation of metastasis-related integrin-α6β4 and MMP-9. Two studies [101,187] reported a reduction in focal adhesions in s-µ*g* (RPM). The lower activation levels of focal adhesion kinase (FAK/PTK2), Rho family proteins (RhoA, Rac1, and Cdc42), and mTORC1 pathway resulted in activation of AMPK pathway and reduced proliferation and metastasis of melanoma cells [101]. Zhao et al. [6] described that s-µ*g* (RPM) also altered the cytoskeleton and nuclear positioning, leading to enhanced apoptosis via suppression of the mTORC1/NF-κB and ERK1/2 pathways. Tan et al. [101] further demonstrated that s-µ*g* (RPM) reduced NADH induction and glycolysis but promoted mitochondrial biogenesis. The RhoA activator CNF-1 (cytotoxic necrotizing factor 1) effectively reversed s-µ*g*-induced changes and effects.

Ivanova et al. [188] used human pigmented, non/low metastatic IF6 and non-pigmented, highly metastatic BLM melanoma cells to study their morphology and cell behavior (cell division, attachment/detachment, migration) when exposed to s-µ*g* (FRC). Although both cell lines proliferated and migrated under reduced gravity without morphologic changes within the first 24 h, preliminary results suggested that cell proliferation is reduced under s-µ*g* (FRC). They also found long-term exposure of human melanoma cells to s-µ*g* (FRC) down-regulated genes for iNOS and guanylyl cyclases (GC) A and B in highly metastatic human melanoma cells and decreased their motility. Most importantly, s-µ*g* (FRC) also reduced NO-sensitive sGC expression in non-metastatic melanoma cells, whose expression and activity are inversely related to tumor aggressiveness [189]. Since GC isoforms sensitive to natriuretic peptides are expressed in cancer cells, including melanoma, and natriuretic peptides are associated with cancers, their finding may suggest that the metastatic potential of melanoma cells can be attenuated in reduced gravity.

Interestingly, colony formation studies on melanoma in s-µ*g* (RPM) indicated a reduced ability of the cancer cells to form colonies [186]. Nevertheless, there have been some efforts and successes in the 3D culture of melanomas using µ*g*. To generate 3D tumor models that mimic the in vivo melanoma tumor microenvironment much better than conventional culture methods, Licato et al. [190] performed the first study with human melanoma cells using the effects of s-µ*g* (RWV). They cultured several primary human melanoma cells on an RWV for 7–8 days and observed tumor spheroids. Immunohistochemical analysis revealed several cell types like the in vivo situation. In an indirect approach, the HARV was used to promote aggregation of keratinocytes, which formed a construct that served as a scaffold for B16F10 cell growth. This 3D model supported melanoma cell growth, proliferation, and synthesis of typical ECM. In addition, the melanoma cells interacted with each other and showed cellular morphological changes [191].

Last year, Przystupski et al. [192] used a novel lab-on-a-chip approach to investigate the effects of short-term s-µ*g* (RPM) on A-375 human melanoma cells. Their experiments confirmed typical µ*g*-related morphological (stress fibers, membrane blebbing, lamellipodia, absence of filopodia) and physiological changes (decrease in cell viability and mitochondrial activity, decreased proliferation, and increase in caspase activity) in cells after short-time exposure to s-µ*g*. The lab-on-a-chip technology can be easily used for studies on various µ*g* platforms, including space on the ISS or satellites. To date, we are not aware of any studies exploring melanoma CSCs in µ*g*.

## 5. Extracellular Vesicles and Microgravity

Cell-cell communication is a vital requirement for any living organism to facilitate the concerted interaction of the various cell types. Even bacterial cells communicate via the so-called quorum sensing within their environment and alter their behavior in response to changes in the setup of their community [193]. For the longest time, the only known methods of cell crosstalk in multicellular systems were via direct cell contact or receptor-based signaling [194]. In recent years, extracellular vesicles (EVs) were discovered as another mode of cellular information exchange and have gained increasing attention since. EVs comprise a family of membrane vesicles that are secreted from the majority, if not all, cells into the extracellular environment and functionally mediate cell-cell communication. Besides micro-vesicles and apoptotic bodies, exosomes, or small EVs, are the major type of extracellular vesicles. Exosomes can be distinguished from other EVs by their biogenesis, size, as well as several surfaces and internal markers [195,196], and are found in virtually all circulating body fluids, including blood, saliva, and urine [197,198,199,200,201]. As their cargo reflects their cell of origin together with environmental influences regarding their protein, nucleic acid, and lipid content, combined with the ease of access, their potential as biomarkers and in clinical applications is apparent.

The effects of µ*g* are widespread and have been described since the early days of space travel, proteomic and genomic changes are manifold [110,202,203,204,205,206]. With the development of new cell biological methods and new hardware systems, it is now possible to conduct cell culture experiments not only under s-µ*g* but also r-µ*g* conditions [87]. Analyzing the cellular changes after short or long-term exposure to microgravity consequently leads to the question of which part cell cross-talk plays in these processes. The more EVs and exosomes, in particular, came into focus as major players in tumor biology, their role in the cellular response to microgravity became an interesting topic to explore. With the recent development of very specific methods for small EV analysis, it is finally possible to explore these questions.

The first group to use exosomes as an analytic tool in µ*g* research were Zanello et al., who hypothesized that the visual impairment and intracranial pressure (VIIP) syndrome described in astronauts returning from long-duration space missions could be studied via the use of an Idiopathic intracranial hypertension (IIH) model because VIIP and IIH share some neurologic and ophthalmologic manifestations. In their 2018 study, the authors describe gene expression signatures obtained from exosomes collected from CSF and plasma in patients with possible signs of IIH. They suggest that inflammatory cytokine-driven processes and immune cell migration are activated when ICP is elevated in IIH patients and that several miRNAs, mainly miR-9 and miR-16, are involved in this response. If similar changes could be seen in astronauts with the VIIP syndrome, IIH could prove to be a good model to study this condition without actual exposure to microgravity [207].

The first study to examine exosomes exposed to r-µ*g* was conducted on endothelial cells. Following preliminary ground-based experiments, EA.hy926 cells were exposed to r-µ*g* onboard SJ-10 Recoverable Scientific Satellite for 3 and 10 d, respectively. The authors’ main focus was on changes in inflammatory pathways and cell growth, but they also reported an enhanced exosome-mediated mRNA transfer [204]. On the same satellite, rat bone marrow-derived mesenchymal stem cells (BMSCs) were examined regarding the effect of r-µ*g* on hepatogenic differentiation. After 3 and 10 d in space, this group similarly described an increase in exosome-mediated mRNA along with improved differentiating capability and maturation of the cells [18].

Zhang and colleagues mapped the systemic fusion analysis of the serum metabolome and the circulating microRNAome in a hindlimb unloading rat model to s-µ*g*. This way, the modulation of neurofunctional signaling pathways should be elucidated. The response of serum metabolites and microRNAs to s-µ*g* was striking, using rabies virus glycoprotein–modified exosomes, the delivery of miR-383-5p inhibited the expression of AQP4 not only in rat C6 glioma cells in vitro but also in the hippocampus in vivo. Particularly, miR-383-5p was significantly increased, and aquaporin 4 (AQP4) decreased in the hippocampus. The authors suggest that bioinformatics could be used to map the crosstalk between the circulating metabolome and miRNAome to better to understand complex biological systems under µ*g* [208].

As part of the NASA Twin Study, a year-long mission on the ISS, blood samples were collected from at the same time points from an astronaut (TW), his identical twin on Earth (HR), and healthy donors. From TW, samples were also collected before and after the flight. The liquid biopsies were profiled in regard to their cell-free DNA (cfDNA) characteristics, including fragment size, cellular deconvolution, and nucleosome positioning. After the year-long mission, a significant increase in cell-free mitochondrial DNA (cf-mtDNA) inflight was shown, and analysis of post-flight exosomes in plasma revealed a 30-fold increase in circulating exosomes and patient-specific protein cargo (including brain-derived peptides). This longitudinal analysis of astronaut cfDNA during spaceflight and the exosome profiles highlights their utility for astronaut health monitoring, as well as cf-mtDNA levels as a potential biomarker for physiological stress or immune system responses related to µ*g*, radiation exposure, and the other unique environmental conditions of spaceflight [209].

EVs and exosomes are of special interest in cancer research since tumor-derived small EVs play a pivotal role in the tumor cells’ immune escape and the education of the tumor microenvironment. Tumor cells are also regularly subjected to s-µ*g* and r-µ*g*, as this may help elucidate many aspects of their development, progression, and metastatic behavior [32,81,90,111,210]. Triple-negative breast cancer (TNBC) has a relatively poor prognosis regardless of therapy, so considerable efforts are being made to reveal the molecular mechanisms and potential diagnostic/therapeutic targets. Chen et al. conducted a study on MDA-MB-231 cells using µ*g* as the sole variable in hopes to identify key molecules that shift tumor cells to a less-aggressive phenotype in TNBC. Additionally, µ*g* can inhibit cancer cell viability, proliferation, metastasis, and chemoresistance, and with a focus on EVs, the authors aim to elucidate the connected signaling pathways. Upon exposure of the cells to a minimum of 96 h of s-µ*g* onto a Gravite gravity controller, the EV release rate decreased in microgravity while the average EV size increased. Furthermore, it was found that EVs may be superior to cells in analyzing differentially expressed proteins, especially ones down-regulated which are generally unidentified or neglected in the analysis of intact cellular contents. Proteomic analysis of both EVs and cells further revealed a significant correlation with GTPases and proliferation of MDA-MB-231 cells in µ*g*.

Lastly, the group around Grimm analyzed the supernatants from thyroid cancer cells, FTC-133, exposed to r-µ*g* for 12 d during a mission on the ISS as part of the Cellbox-1 Experiment on the changes in exosome release, particle size, and tetraspanin surface expression [105]. In a follow-up analysis, these exosomes were scanned regarding their miRNA content [106]. The initial studies showed increased release of these small EVs in the samples flown to space (FMs) compared to their ground controls; the size distribution reflected these results. The expression of the tetraspanins CD9, CD63, and CD81 followed the same pattern, with CD63 being significantly higher expressed in the FM samples [105]. An array scan of a total of 754 miRNA targets revealed 119 differentially expressed miRNAs, 19 of which were elevated, the remaining 100 decreased in direct comparison of FMs to GMs. Of these differentially expressed miRNAs, 23 have been described in the pathogenesis of various thyroid cancers previously, and three of the downregulated miRNAs, namely hsa-miR-429, hsa-miR-128-3p, and hsa-miR-199, were analyzed further to confirm the array results [106].

All of the above studies show the possible opportunities that EV research in µ*g* offers in search of solutions to minimize the effects of µ*g* on space travelers and the quest to find new therapies in a variety of diseases.

## 6. Multicellular Tumor Spheroids as a Metastasis Model

The µ*g*-environment induces the formation of 3D aggregates, particularly MCS and organoids. Culturing cells under µ*g*-conditions promotes the development and the production of large well-differentiated aggregates. Both r- and s–µ*g* platforms provide a new technology for the large-scale production of organoids and spheroids for cancer research and drug testing. In the last 30 years, a large number of studies have focused on the behavior of cells using different tissue types exposed to altered gravity. In 1993, Lewis et al. reported about the use of µ*g*-bioreactors for engineering in vitro models to investigate steroid-mediated secretion of EGF by normal salivary gland cells and for the investigation of cancer and other salivary gland disorders [211].

**Figure 4:**
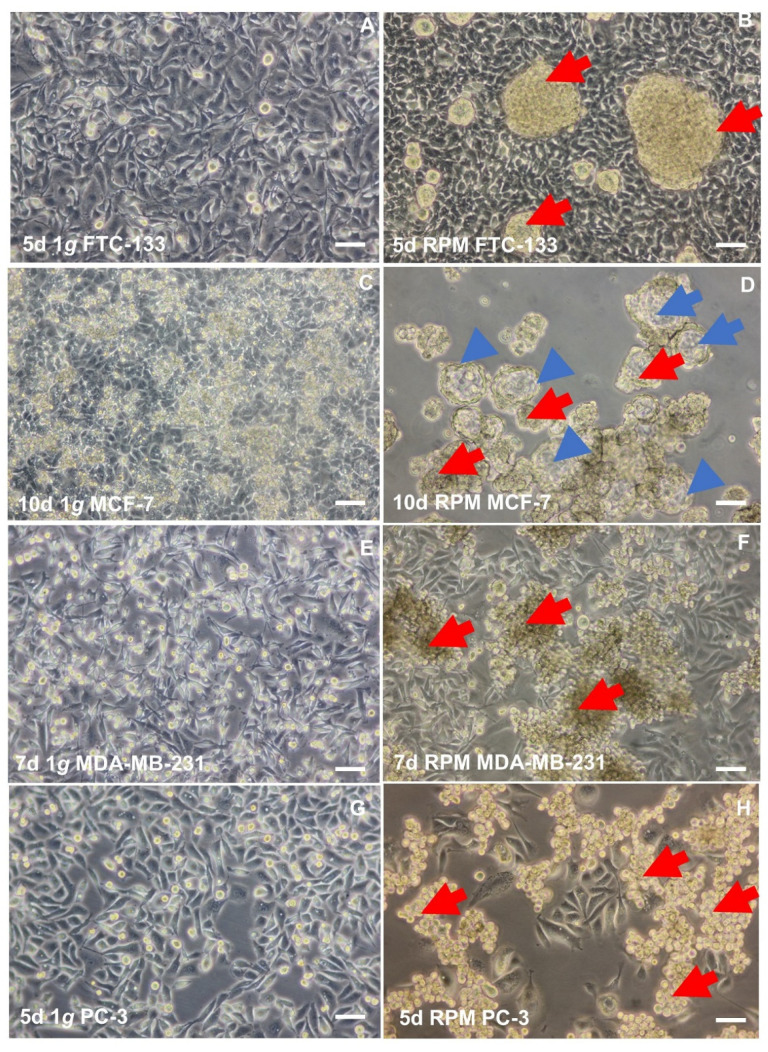
Phase contrast microscopy: (**A**) 5d, 1*g* FTC-133 TCC, (**B**) 5d RPM FTC-133 cells, MCS are visible (red arrows); (**C**) confluent and overgrown MCF-7 BC cells after 10 d at static 1*g* conditions, (**D**) 10-d RPM-exposure of MCF-7 revealed compact MCS (red arrows), but also glandular structures (blue arrows); (**E**) 7 d, 1*g* MDA-MB-231 BCC, (**F**) 7-d exposure of MDA-MB-231 triple negative BCC revealed 3D MCS; (**G**) 5 d, 1*g* PC-3 PCC and (**H**) compact 5 d MCS of PC-3 cells on the RPM (red arrows); magnification ×100.; scale bars: 50 µm.

The majority of the investigated cells from different tumor types, such as, TC, BC, CRC, HCC, LC, and PC grew in form of 3D spheroids after different lengths of µ*g*-exposure. Some MCS exhibited a great resemblance to metastases. BC MCS scaffold-free engineered on the RPM showed a compact structure after 24 h. Others also revealed a glandular shape after long-term exposure to s-µ*g* [62]. TCC, BCC, LCC and PCC cultured on the RPM formed scaffold-free 3D aggregates [31,61,62,63,64,118,178] suitable for future knock-in/knock-out experiment or pharmacological interventions (Figure 3B and Figure 4B,D,F,H).

Compared to 1*g*-MCS, which were generated using, among others, ‘hanging drop,’ ‘liquid overlay,’ or the ‘spinner flask’ technique, MCS generated under µ*g* were characterized by a lack of necrosis in their center [61]. Healthy thyroid cells can also be cultivated on the RPM for several weeks under s-µ*g* [61,114].

As reviewed in Section 4.5, other research groups used the NASA-developed RWV for tissue engineering of organoids. Liver-based cell organoids were constructed in an RWV bioreactor. Afterward, they were inoculated with colon carcinoma cells (HCT116) together with hyaluronic acid and gelatin-based microcarriers inside the RWV in order to create liver-tumor organoids for in vitro modeling of liver metastasis [141]. The WNT pathway seems to be involved in organoid formation. Manipulating the WNT pathway with an agonist and antagonist showed significant changes in sensitivity to the anti-proliferative drug 5-fluoruracil (5-FU) [141].

CRC cells (HCT116) have been co-cultured with primary human hepatocytes and mesenchymal stem cells (MSC) and seeded on hyaluronic acid-based microcarriers, loaded with liver-specific growth factors and ECM components in the RWV. They grew as 3D aggregates. The MSC appeared to drive self-organization and formation of a stromal-like tissue surrounding the tumor foci and hepatocytes [142]. Exposure to the chemotherapeutic agent 5-FU showed dose-dependent cytotoxicity. These findings demonstrate the suitability of liver tumor organoids for cancer progression and pharmacological drug testing [142] and that they could also be used in the future for pharmacological tests or co-culture experiments (angiogenesis models), for example, in order to reduce the number of animal experiments.

**Figure 5:**
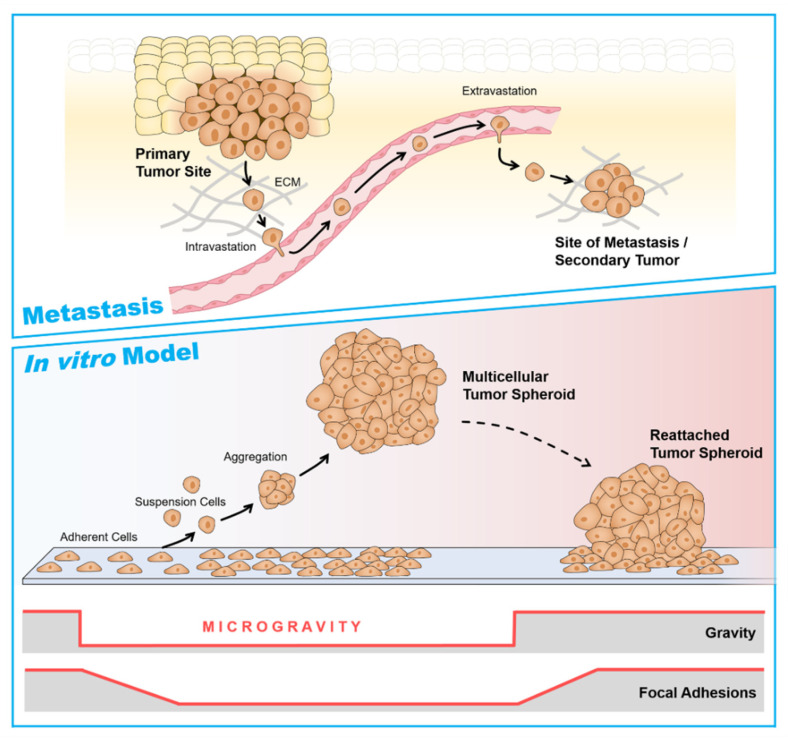
Schematic representation of the µ*g*-induced in vitro metastasis model. When exposed to µ*g*, adherent cancer cells downregulate focal adhesions. The cells detach and micro-metastasis-like tumor spheroids are formed. When gravity is restored, the spheroids reattach on their substrate.

The cells growing in the form of MCS not only exhibit a different phenotype but also behave differently than those growing in flat monolayers on the bottom of the cell culture flask under 1*g*-conditions. They are well-differentiated and more closely mimic conditions in the human body. For example, HCC (MHCC97H) cells exposed to the RWV grew as spheroids and mirrored features of HCC in vivo [151]. Therefore, the spheroid and organoid models have become an invaluable tool for studying metastases and developing new strategies for cancer treatment through drug targeting (Figure 5) [96,114,142].

Weightlessness deeply influences processes such as apoptosis and structural changes in the cytoskeleton and extracellular matrix that affect cell growth [79]. TCC in space formed MCS, and the response to µ*g* was mainly anti-proliferative [65]. This result detected in space-flown FTC-133 cells was also confirmed by other cancer cells exposed to s-µ*g* [179,180]. The ERK/RELA pathway was identified as a major µ*g*-regulatory pathway involved in spheroid formation in TCC and PCC [65,118]. At the moment, various factors are known to support the 3D growth of cancer cells grown in space or under s-µ*g*. Table 1 summarizes the published genes and proteins involved in the biological processes of angiogenesis and metastasis of TCC.

Findings from µ*g*-research could be used to complement conventional cancer research and to localize the cellular changes that lead to carcinogenesis and promote progression and metastasis. This knowledge, in turn, could lead to novel therapies that could improve patients’ quality of life or potentially contribute to new preventive countermeasures

## 7. Current Knowledge about Proteins as Candidates for Future Targeted Tumor Therapy

Using proteomics, in 2010, we were able to identify 37 proteins that had not been described in the thyroid. Examples are copine-1, plastin-2, septin-11, or transgelin-2. Plastin-2, playing a crucial role in the development of thyroid tumors, and ISG15 (interferon-stimulated gene 15 or interferon-induced 17 kDa protein precursor) are of great interest. [103]. The meaning of ISG15 in TC is not yet clear. It acts as an extracellular cytokine but is also involved in protein synthesis intracellularly. The ISG15 gene was first found in the Sino-German SimBox/Shenzhou-8 space experiment (Thyroid Cancer Cells in Space) and was detected in later proteome analyses [212]. ISG15 is known to disrupt the F-actin cytoskeletal structure and focal adhesions as well as promote motility in human ZR-75-1 breast cancer cells [213]. Therefore, future studies focusing on ISG15 in other cancer cells exposed to µ*g* should be performed.

R-µ*g* has an impact on the differentiation of malignant tumors. During the Shenzhou-8/SimBox space mission, when thyroid cancer cells were cultured in space for 10 d, the gene expression pattern was altered. The microarray analysis revealed 2881 significantly altered transcripts after 10 d on the RPM or in space. The genes of several biological processes were differentially expressed. Genes and proteins involved in regulating cancer cell proliferation and metastasis, such as *IL6*, *IL8*, *IL15*, *OPN*, *VEGFA*, *VEGFD*, *FGF17*, *MMP2*, *MMP3*, *TIMP1*, *PRKAA,* and *PRKACA*, were similarly regulated under s-µ*g* and r-µ*g* [88]. In particular, the growth factors *VEGF*, *EGF*, and the interleukins *IL6* and *CXCL8* have been identified as candidate genes [87,88] to be targeted in the future.

The NFκB p65 pathway is a key player in the progression and metastasis of various cancer types [214]. NFκB is a known key player in the development of malignant tumors. It contributes to the spheroid formation of MCF-7 BCC and FTC-133 TCC exposed to the RPM and can be targeted with dexamethasone (DEX) [96,113]. With the development of novel technology, the inhibition of NFκB by a variety of inhibitors will result in future personalized treatment strategies.

Reducing or even preventing metastasis by targeting one or more of these factors might improve patients’ survival and cancer therapy. Therefore, similar studies on other types of tumors are needed urgently. With the investigations on, e.g., TCC and BCC, this work is now moving from the exploratory phase to functional analysis of individual candidate genes previously identified.

Dexamethasone (DEX) was able to suppress spheroid formation in follicular FTC-133 cells and MCF-7 cells cultured on the RPM. DEX dose-dependently inhibited the growth of 3D cell aggregates. Several factors involved in cancer progression of TCC like autocrine signaling, proliferation, epithelial-mesenchymal transition, and anoikis were measured. Wnt/β-catenin signaling, *NFKB2*, *VEGFA*, *CTGF*, *CAV1*, *BCL2 (L1)*, or *SNAI1* were clearly altered by DEX. These results suggest a regulatory network of MCS formation involving additional signaling pathways or factors influenced by DEX [96].

Further studies with the Src inhibitor PP2 and an anti-E-cadherin antibody also demonstrated their impact on the spheroid formation of MCF-7 cells. While the use of PP2 completely inhibited MCS formation, the E-cadherin antibody induced the formation of 3D aggregates. Neither agents influence cell viability. The survival rate of the cells was 100% [114].

Based on these results, further pharmacological studies are planned. The drugs and antibodies that appear suitable based on earlier publications are listed in Table 5.

The majority of these studies have been performed by our team. We studied the behavior of BCC and TCC earlier, and for the last two years, we have studied PCC under s–µ*g* conditions using the RPM as well. Therefore, it is necessary to investigate various cancer types in space and under conditions of different µ*g*-devices in order to find common mechanisms for metastasis and the key proteins responsible for this, which can be inhibited by targeted therapy. Importantly, we worked with scaffold-free MCS models and used 2D cell monolayer cultures in cell culture flasks or special flight hardware when we cultured the cancer cells in space and on the RPM or clinostat. In the case of gastrointestinal system neoplasms, researchers have always started with single floating cells that aggregate once inside the RWV or RCCS device. Therefore, future studies should be performed to investigate CRC or liver cancer cells on the RPM or in space, followed by multi-OMICS analyses to find new interesting targets.

## 8. Multi-Omics Analyses, a New Perspective for Microgravity-Related Cancer Research

In multi-omics analyses, data sets from different omics domains are integrated to reach a common conclusion. In particular, numerous combinations of genomic, transcriptional, proteomic, metabolomic, and epigenomic datasets have been published in the last 10 years. However, only a few in the field of cancer research in space had been performed and discussed in earlier chapters in this review [88,90,103,106,114,216].

**Figure 6:**
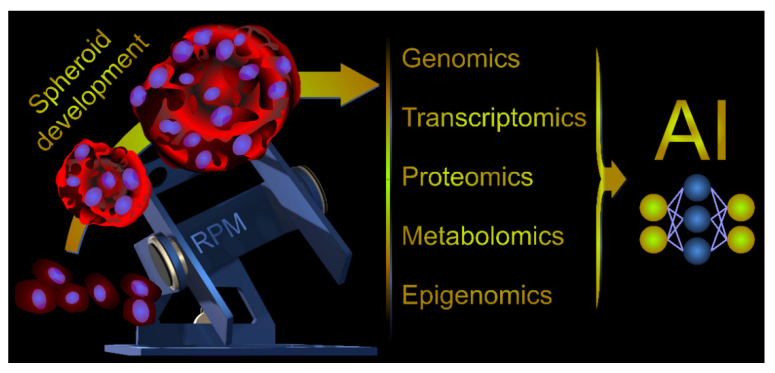
Illustration of a Multi-Omics workflow in cancer research. Starting with the MCS development enabled by µ*g*, the subsequent omics analyses using NGS technology and mass spectrometry generate multi-omics data sets that are finally processed integratively in AI-based analyses. The graphic under the term AI is a simplified representation of an artificial neural network.

Aristotle’s “Therefore, the syllable is some particular thing; not merely the letters, vowel and consonant, but something else besides.” Ref. [217] involuntarily describes the multi-omics intention to capture the information flow between the individual omics levels to understand the underlying complex biological processes better. Cancer research [218] and especially precision medicine [219,220] benefit from the rapid development of omics technologies. High dimensionality and heterogeneity of the data often require analysis techniques beyond simple test statistics, so AI-based analyses are on the rise, particularly machine learning and deep learning methods [221,222,223,224] (Figure 6).

Multi-omics studies related to µ*g* and cancer are currently rare, but the open-access NASA GeneLab provides advanced multi-omics analysis capabilities related to spaceflight response [225]. Fujita et al. used multiple spaceflight mouse RNAseq datasets from the GeneLab for re-analyzing and found that the clock gene expressions of spaceflight mice differed from those of ground controls and that spaceflight caused asynchrony of clock genes between tissues [226]. Previous studies have shown that circadian rhythm disturbances are a serious cancer risk [227,228,229].

The twin study by Bezdan et al. examined the effects of the one-year ISS mission of a fifty-year-old twin on cell-free DNA (cfDNA), cell-free mitochondrial DNA (cf-mtDNA), and exosomes [209]. Concentration, fragment length distribution, mutations, and methylation levels of cfDNA can be used as indicators of cancer and other physiological abnormalities [230,231]. The twins did not show cfDNA concentration differences. However, slightly increased cfDNA concentrations before and after the flight and a change to longer cfDNA fragment lengths were detected in the twin on the ISS mission [209]. A change to shorter cfDNA fragment lengths may be one of the indicators of cancer. A potential biomarker of physiological stress is the increased amount of cf-mtDNA relative to cfDNA, especially at the beginning of the flight. Counting exosomes three years after the flight showed a significantly increased number of particles of the ISS return, mass spectrometry showed a similar protein number, but an enrichment of proteasome pathway proteins in the ISS return. Likewise, Bezdan et al. found an association of the exosome to the 20S proteasome three years post-return. Elevated 20S proteasome levels are unspecific indicators for stress response but have also been observed in various blood cancers and solid tumors [232].

In conclusion, multi-omics analyses open new possibilities for the study of spaceflight response and µg-based cancer research. However, standing at the beginning of this development, we are sure that in a few years, especially in cutting-edge research, multi-omics will have a high value.

## 9. Methods

**Figure 7:**
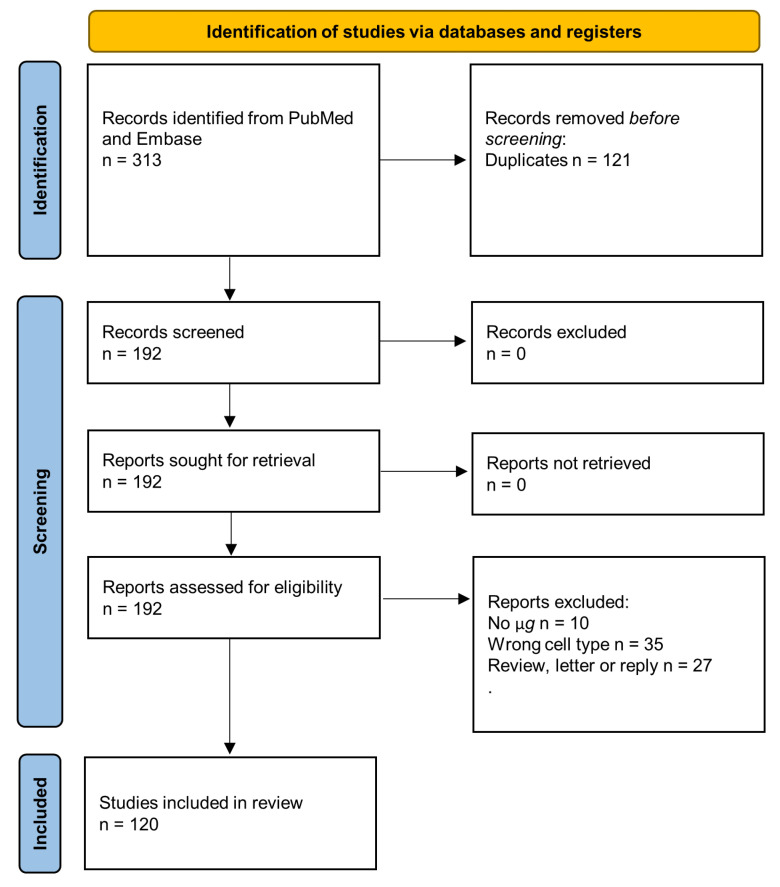
PRISMA 2020 flow diagram: results of the literature search (different types of cancer cells exposed to microgravity/space) for this comprehensive review.

We searched the PubMed (available at https://pubmed.ncbi.nlm.nih.gov/ accessed on 15 January 2022) and Embase (available at https://www.embase.com accessed on 1 February 2022) literature databases using the search terms “microgravity” or “simulated microgravity” or “real microgravity” or “weightlessness” or “spaceflight” or “clinostat” or “clinorotation” or “Random Positioning Machine” together with “exosomes” or “extracellular vesicles EV” or “breast cancer cells” or “omics cancer” or “gastrointestinal cancer cells” or “hepatic cancer cells” or “colon cancer” or “gastric carcinoma” or “pancreatic neoplasms” or “thyroid cancer cells” or “lung cancer” or “lung cancer stem cell” or “melanoma” or “skin cancer”. The resulting PRISMA (Preferred Reporting Items for Systematic Reviews and Meta-analyses) flow diagram is shown in Figure 7.

## 10. Conclusions

Experiments with cancer cells performed in space on the ISS or under s-µ*g*-conditions using devices suitable and acknowledged from ESA and NASA to create µ*g* conditions on Earth, known as ground-based facilities (Section 2), belong to a newly evolving area of research in cancer biology [233].

A large number of studies have demonstrated how a short- and long-term exposure to r- and s-µ*g* influences, among other processes, differentiation, growth behavior, migration, proliferation, survival, apoptosis, and adhesion, all relevant to cancer [121].

In this comprehensive review, we have discussed the available literature about various types of cancer cells studied under conditions of weightlessness and focused on the formation of 3D spheroids or organoids.

A common result is that r- and s-µ*g* altered the growth behavior of human cancer cells in vitro. A large number of publications reported about the formation of 3D aggregates, also called (multicellular) spheroids, clumps, or tissues in µ*g* (Table 1, Table 2 and Table 3 and Figure 4). The 3D model systems ‘Multicellular Spheroids’ and organoids are often used as metastasis models for various types of cancer. These models are important for drug testing in pharmacology or can be used for co-cultures to study angiogenesis or metastasis. Studies focusing on the underlying mechanisms for 3D formation can increase our knowledge about in vivo cancer progression, epithelial to mesenchymal transition (EMT), and metastasis.

Various factors seem to be involved in 3D spheroid formation. Proteomics studies found more than 5900 different proteins in MCS and adherent static 1*g*-control cells. Proteins proposed for spheroid formation are ASAP1 and stabilize ISG15 in cancer cells [97]. Moreover, FTC-133 TCC growing in the form of two phenotypes (adherent monolayers or MCS) after RPM-exposure incorporate vinculin, paxillin, focal adhesion kinase 1, and adenine diphosphate (ADP)-ribosylation factor 6 in different ways into the focal adhesion complex [97].

Furthermore, the E-cadherin suppressor Src was found in spheroids combined with a decreased amount of E-cadherin. Indeed, blocking E-cadherin prevented spheroid formation in MCF-7 cells [114]. The balance of proteins modulating E-cadherin expression regulates MCS formation on the RPM. NFκB, which also represses E-cadherin [234], was elevated in the nuclei of MCF-7 MCS cells [113]. In addition, cytokines like IL-6 and IL-8 are involved in the spheroid formation of PCC exposed to the RPM [118].

Organoids from cancer cells bio-fabricated in space followed by a proteomics study had been performed by Cristobal et al. [235] tissue in CRC patients. The authors demonstrated that these organoids recapitulate diversity among patients and CRC features [235].

Proteomics studies of various cancer cell types indicated several factors and pathways involved in cell adhesion and MCS formation, such as MAPK, and PAM, Wnt, VEGF, MAPK, and PAM signaling, and factors like among others NFκB, TGF-β, FAK/RhoA/Rock or β-catenin [63,66,96]. TCC investigated during the CellBox-2 ISS space mission grew adherently and in the form of MCS in space. ERK/RelA was identified as a major µ*g*-regulatory pathway [65].

A recent publication about the secretion of exosomes of TCC in space demonstrated differences in the number of released exosomes, as well as in the distribution of subpopulations concerning their surface protein expression. Population changes regarding the tetraspanin surface expression were determined [105]. Exosome studies will increase the current knowledge of the changes and adaptations of cancer cells to µg-exposure. Additionally, this novel approach may offer new biomarkers and possible therapies for different types of cancer and other diseases. Analyzing the exosomal microRNA composition after spaceflight might elucidate some of the proteomic changes, which had been reported earlier [106].

The fields of space medicine, gravitational biology, and cancer research in space support the search for new proteins in different types of cancer cells. As µ*g* provides environmental conditions organisms never encounter on Earth, it may provide special conditions that are helpful to detect changes in proteins that may become new targets for tumor drug development. Further μ*g*-studies should be performed to investigate tumor growth, mechanisms of progression, and metastasis. The CANCEROIDS ISS space mission (2023/2024) will study eGFP-vinculin and mCherry-β-catenin-transfected MCF-7 BCC for 10 d in space and will be carried out with a focus on focal adhesions in µ*g* and spheroid formation, using ‘live cell imaging’ with the FLUMIAS microscope [85,102] on board the ISS or in the future on sounding rockets and parabolic flights.

## Figures and Tables

**Table 1:** Overview of Results of Studies with Thyroid Cancer Cells.

Cell Line	Biological Process	Genes/Proteins/Pathways Major Results	Microgravity	Reference
FTC-133	Adhesion	VCAM1	Space–CellBox-1	[89,90]
		*VCL, PXN, ICAM1*	Space–CellBox-2	[65]
FTC-133	Angiogenesis	*VEGFA, VEGFD, FLK1*	Space–SimBox	[88]
		VEGF-A	Space–CellBox-1	[89]
		Angiopoetin-2	Space–CellBox-2	[65]
		*VEGFA*, VEGF-A	Space–CellBox-2	[104]
FTC-133	Caveolae	CAV1	Space–CellBox-1	[89,90]
		*CAV1*	Space–CellBox-2	[65]
FTC-133	Extracellular Matrix	*SPP1, MMP2, MMP3, TIMP1*	Space–SimBox, RPM	[88]
		TIMP1, MMP3	Space–CellBox-1	[89]
		*COL1A1, ITGB1*	Space–CellBox-2	[65]
FTC-133	Cytokines	*IL6, CXCL8, IL15*	Space–SimBox, RPM	[88]
		IL6, IL8, IL7, IL18, MCP1, MIP-1 beta	Space–CellBox-1	[89]
		*IL6*	Space–CellBox-2	[65]
ML1, RO82-W1		IL6, MCP1	RPM, Clinostat	[31]
		IL6, IL8	1*g* Liquid-overlay	
FTC-133	Cell Signaling	*ERK1/2, RELA*	Space–CellBox-2	[65]
FTC-133	Protein Kinases	*PRKAA, PRKACA*	Space–SimBox, RPM	[88]
FTC-133	Growth Factors	*EGF, CTGF, FGF17*	Space–SimBox, RPM	[87,88]
		*EGFR*	Space–CellBox-2	[65]
FTC-133	Cytoskeleton	*ACTB*, *TUBB*, F-actin	Space–TX52	[85]
ML1		*ACTB*, *KRT80*	PFC	[82]
		Cytokeratin, vimentin, tubulin	PFC	[82]
FTC-133	Exosomes, Exosomal miRNA	CD9, CD63, CD81	Space-CellBox-1	[105]
		Array scan of a total of 754 miRNA targets revealed more than 100 differentially expressed miRNAs: miR-199 family	Space-CellBox-1	[106]

**Table 2:** Genes and proteins, involved in BCC spheroid formation under microgravity conditions.

Cell Line	Biolog. Process	Genes/Proteins/Pathways Major Result	Microgravity	Ref.
MCF-7	Cytoskeleton	Upregulation of *KRT8*, *RDX*, *TIMP1*, *CXCL8* mRNAs, downregulation of VCL, reduced E-cadherin protein and rearrangement of F-actin and tubulin	r-μ*g*/TEXUS SR & PFC	[110]
MDA-MB231	Cell adhesion	Upregulation of *ICAM1*, V*CAM1*, *CD44* and down-regulation of NFκB-p65 and annexin-2 protein.	r-μ*g*/PFC	[111]
MCF-7	ECM, Cell cycle, Proliferation	Loosely organized perinuclear cytokeratin network, arrested cell cycle and decreased proliferation	r-μ*g*/spaceflight	[112].
MCF-7	Cytoskeleton, Mitosis	Altered microtubule structure, prolonged cell cycle	r-μ*g*/spaceflight	[127]
MCF-7	MCS Cytoskeleton	*ACTB*, *TUBB*, *EZR*, *RDX*, *FN1*, *VEGFA*, *FLK1*, *CASP9*, *CASP3*, *PRKCA* mRNAs were downregulated in 5 d-MCS, duct-like and compact MCS	s-μ*g/*RPM	[62]
CRL-2351	Cell reparation and adhesion, MCS	*BRCA1* increased, *KRAS* decreased in AD cells; *VCAM1* upregulated, *VIM* downregulated in µ*g*	s-μ*g/*iRPM	[115]
CRL-2351	Morphology and gene expression, MCS	Upregulated *RHOA* gene and over expressed *MAPK1* gene and protein	s-μ*g/*RPM	[116]
MCF- 7	MCS formation and adhesion	Decreased E-cadherin in MCS, PP2 prevented MCS formation	s-μ*g*/RPM	[114]
MCF-7	MCS formation, apoptosis	Upregulation o*f ANXA1*, *ANXA2*, *CTGF*, *CAV2*, *ICAM1*, *FAS*, *CASP8*, *BAX*, *TP53*, *CYC1*, and *PARP1* in MCS, upregulated apoptosis related protein p53, *CYC1*, *PARP1*, *FAS*, *CASP8*, and *ANXA1*	s-μ*g/*RPM	[83]
MDA-MB231	Phenotypic switch	G_2_/M inhibited and cyclin D1 decreased	s-μ*g*/RPM	[64]
MCF-7, MDA-MB231	MCS formation	Vinculin and β-catenin are critical to form MCS	s-μ*g*/RPM	[66]
MCF-10A, MCF-7	Apoptosis	increased AKT and ERK pathway activity, decreased apoptosis	s-μ*g*/RPM	[119]
MDA-MB231	Cell cycle apoptosis	Increased lysosomal vesicles, cyclin D3, decreased Bcl-2 and MMP9 proteins.	s-μ*g*/RCSS	[124]
MCF7	Metastatis ability	Cell invasion and migration decreased	s-μ*g*/MG-6C clinostat system	[109]
MDA-MB 231	dysregulation extracellular vesicle	Proteomics show significant correlation with GTPases and proliferation	s-μ*g/*Gravite	[126]

**Table 3:** Genes and proteins involved in prostate cancer cell spheroid formation under microgravity conditions.

Cell Line	Biological Process	Genes/Proteins	Microgravity	Reference
DU145	Cytoskeleton	Cytokeratins-8 and -18, actin, vimentin	s-µ*g*: high aspect ratio vessel (HARV)	[130]
DU145	Regulatory and matrix proteins	EGF, EGF receptor, TGF-β1, TGF-β receptor, collagen IV and laminin	s-µ*g*: (HARV)	[131]
DU145	Transduction-second messenger	DAG, ceramide, PA, PEt, choline, AA and cAMP	s-µ*g*: (HARV)	[132]
LNCaP	Prostate specific peptidase	PSA	s-µ*g:* (HARV)	[133]
PC-3	Cell adhesion molecules	CD44 and E-cadherin	s-µ*g*: (HARV)	[135]
PC-3	Epithelial marker	cytokeratin VIII	s-µ*g*: (HARV)	[135]
PC-3	Collagen deposition	collagen IV	s-µ*g*: (HARV)	[135]
PC-3	VEGF signaling	*VEGFA*, *FLK1*, *RAF1*, *SRC1*, *AKT1*, *MTOR*, *MAP2K1*, *ERK2*, *LCN2*, protein supernatant: NGAL, VEGF	s-µ*g*: RPM	[63]
PC-3	Collagen deposition	*LAMA3*, *LAMB2*, *FN1*	s-µ*g*: RPM	[63]
PC-3	Focal adhesion	*CDH1*	s-µ*g*: RPM	[63]
PC-3	Cytokines	IL-1α, IL-1β, IL-6 and IL-8	s-µ*g*: RPM	[118]

**Table 4:** Overview of results of studies with gastrointestinal cancers exposed to µ*g*.

Cell Line	Biological Process	Genes/Proteins/Pathways Major Results	Microgravity	Reference
**Colorectal Cancer Cells**				
HT-29, HT-29KM, Co-culture with normal human colonic fibroblasts	Differentiation	proliferation at an accelerated rate, organizing themselves into 3D MCS (1.0–1.5 cm), signs of a well-differentiated colon tissue	RWV	[137]
HT-29KM CCL 188 KM-12c and MIP-101	Cell adhesion	µ*g* does not alter epithelial cell adhesion	RWV	[138]
MIP-101	Proliferation, differentiation	The petri and RWV cultures continued to proliferate the full 14 d. Induced expression of CEA	RWV with 5 mg/mL Cytodex 3 microcarrier beads	[139]
MIP-101	Differentiation, Apoptosis, Proliferation	Rotation appears to increase apoptosis and decrease proliferation, whereas static 3D cultures in either unit or microgravity have less apoptosis, and reduced rotation in microgravity increases CEA expression	on Teflon-coated non-adherent surfaces (static 3D) or RWV either in r-µ*g* low-earth orbit or in unit gravity on the ground (3D 1*g*)	[140]
HCT-116	3D liver metastasis model with CRC cells Drug testing with 5-FU	In 2D they displayed an epithelial phenotype, and only after transition to the organoids did the cells present with a mesenchymal phenotype. WNT pathway might be involved in the phenotypic changes In vitro 3D liver-tumor organoid model for metastasis growth and suitable for drug testing	RWV	[141]
HCT116	3D spheroids Metastasis model for drug testing-5-FU	Host-liver CRC- spheroids composed of primary human hepatocytes, MSC and HCT116 cells The presence of MSC appeared to drive self-organization and formation of a stroma-like tissue surrounding the tumor foci and hepatocytes.	RWV	[142]
DLD1, HCT116 SW620	Apoptosis 3D aggregates (clumps)	Apoptosis under s-µ*g* Upregulation of the tumor suppressors *PTEN* and *FOXO3* mRNAs leading to *AKT* downregulation and apoptosis induction Clumps formed in µg showed elevated hypoxia and mitochondrial membrane potential	RCCS-HARV	[143]
HCT 116	Stemness regulators, differentiation	upregulation of markers like CD133/CD44, YAP nuclear localization and increase the number of polyploid giant cancer cells, Yamanaka factor upregulation	RCCS-HARV	[71]
Caco-2 cells	Proteomics	38 and 26 proteins differently regulated by simulated microgravity after 48 and 72 h lower NF-kB basal activation in s-µ*g* conditions	2D clinostat	[144]
LS180	Tissue engineering, phytomedicine testing	3D LS180 cell mini-tumors, suitable for drug testing	2D Clinostat	[145]
**Hepatocellular Carcinoma Cells**				
HepG2	3D formation Gene expression	Early stage of 3D assembly: changes in the expression of 95 genes (overexpression of 85 and downregulation in 10)	RCCS	[148]
HepG2	Gene expression	139 genes significantly altered in s-µ*g*	RCCS	[149]
HepG2	MCS formation, cytoskeleton, Gene expression	MCS up to 100 µm in diameter within 72 h and up to 1 mm with long-term culture. MCS: cortical actin organization RWV MCS: upregulation of metabolic and synthetic genes liver-specific functions of cytochrome P450 activity and albumin production are higher in the MCS	RWV	[150]
MHCC97H	MCS formation, Morphology Nude mice model	MCS: mirrored clinical pathological features of HCC in vivo: morphology, ultrastructure, protein production and secretion, glucose metabolism, tissue-specific gene expression, and apoptosis. Xenografts into livers of nude mice resulted in tumorigenesis and distant metastasis	RWV	[151]
MHCC97H	Co-culture of CRC cells and liver fragments Invasion simulation	time-course analysis showed dynamic gene alterations: *MMP2*, *MMP7*, *MMP9*, *CD44*, *SPP1*, *CXCR4*, *CXCL12*, and *CDH1*. Increase in vitronectin, Met, clusterin, ICAM1, GSN proteins	RWV	[152]
MHCC97H, Hep3B	Metastasis—low and high potential, gene expression	Differences between two HCC MCS types in gene expression patterns of adhesion molecules, matrix secretion, invasion etc.	RWV	[153]
HepG2	Apoptosis, cis-diamminedi-chloroplatinum (CDDP)	µ*g* altered CDDP sensitivity through activation of caspase-3 by p53-independent mechanism	Gravite (3D clinostat)	[154]
HepG2/C3A	3D model for genotoxicity testing of chemicals	21-day old MCS: higher basal expression of genes encoding metabolic enzymes compared to monolayer culture. Sensitive and promising in vitro model for genotoxicity and environmental studies.	dynamic clinostat bioreactor system (CelVivo BAM/bioreactor)	[155]
**Gastric and Pancreatic Cancer Cells**				
HGC-27	Metabolomics	A total of 67 differentially regulated metabolites were identified, including upregulated and downregulated metabolites. Phosphatidyl ethanolamine, phosphatidyl choline, arachidonic acid and sphinganine were significantly upregulated in s-µ*g.* sphingomyelin, phosphatidyl serine, phosphatidic acid, L-proline, creatine, pantothenic acid, oxidized glutathione, adenosine diphosphate and adenosine triphosphate were significantly downregulated	RCCS	[156]
NOR-P1	3D tissues, apoptosis	s-µ*g*: NOR-P1 cells showed greater numbers of mitotic, cycling (Ki-67-positive), nuclear factor-kappa B-activating cells, and a lower number of apoptotic cells compared to 1*g*	RCCS-4D	[157]

**Table 5:** Substances acting on the proteins detected in microgravity studies.

Pharmacological Agent and Drugs	Target Protein	References
PP2 (4-amino-5-(4-chlorophenyl)-7-(dimethylethyl) pyrazolo [3,4-d] pyrimidine)	Proto-oncogene tyrosine-protein kinase Src	[114]
Daidzein	Caveolin-1	[89,97]
Camptothecin	Ubiquitin-like protein ISG15	[212]
SP600125	Mitogen-activated protein kinase 8/JNK1	[97]
Dexamethasone, BAY 11-7082	NFκB p65	[96,97,113]
GSK2256098, MPAP	Focal adhesion kinase 1	[97]
MT189	Paxillin	[97]
Cetuximab, Panitumumab, Sym004	EGF receptor	[65]
Interleukin-6 Inhibitor (Siltuximab), Tocilizumab	Interleukin 6, IL-6 receptor	[65,81,88]
HuMax-IL8 (BMS-986253) antibody, CXCL8-IP10 (Analogue), Reparixin	CXCL8, CXCL8 receptor	[65,81,88]
AKT Inhibitor, Ipatasertib	AKT	[63,119]
mTOR inhibitors	mTOR	[63]
Curcumin	HMOX-1	[113]
TM5441	Plasminogen activator inhibitor 1	[113]
UK370106	Stromelysin, (MMP3)	[95]
Monoclonal antibody	Integrin-ß1, Fibronektin, CD44, E-cadherin, ICAM-1, VEGF	[63,81,88,113,114,215]
